# MN•CS•IO System: Cross‐Species Study for HBB Function in High‐Altitude Hypoxia Adaptation

**DOI:** 10.1002/advs.78002

**Published:** 2026-09-28

**Authors:** Wenqiao Hui, Lin Mao, Binggang Li, Hanyue Kang, Zhengquan Tang, Sheng Chen, Hui Yang, Yu Qiao, Huiqian Ding, Yuhuan Wang, Min Li, Junjie Hu, Shoudong Ye, Xin Wang, Xiaobin Xu, Yupeng Wang, Tana Wuren, Qian Ban, Baowei Zhang

**Affiliations:** ^1^ Anhui Provincial Key Laboratory of Livestock and Poultry Product Safety Institute of Animal Husbandry and Veterinary Medicine Anhui Academy of Agriculture Sciences Hefei China; ^2^ Center for Stem Cell and Translational Medicine School of Life Sciences and Medical Engineering Anhui University Hefei China; ^3^ Key Laboratory of Advanced Civil Engineering Materials of Ministry of Education Key Laboratory of D&A for Metal‐Functional Materials School of Materials Science & Engineering Tongji University Shanghai China; ^4^ Research Center for High Altitude Medicine Qinghai University Xining China; ^5^ Center for Micro Nano Systems School of Information Science and Technology (SIST) Fudan University Shanghai China; ^6^ College of Resources and Environment Anhui Agricultural University Hefei China

**Keywords:** gene delivery, HBB, high‐altitude, hypoxia adaptation, Qinghai‐Tibet Plateau animals

## Abstract

The genetic basis of high‐altitude hypoxia adaptation remains largely elusive, particularly the role of HBB. Here, we developed an integrative mesh‐nanorod (MN) cell sorting, chitosan nanoparticle (CS) delivery, and in vivo intraosseous (IO) injection (MN•CS•IO) system to functionally investigate HBB in cross‐species high‐altitude hypoxia adaptation. Using this MN•CS•IO platform, we transplanted high/low‐altitude species HBB‐modified lineage‐negative (Lin^−^) cells into murine models, achieving 95.59% transfection efficiency and 8.15% HBB relative expression, and subjected them to simulated and natural Qinghai‐Tibet hypoxia conditions. High‐altitude HBB recipients exhibited downregulated Rragd expression, activated oxygen transport and hematopoietic lineage pathways, improved behavioral performance, and neuroprotection against hypoxia‐induced damage, compared with low‐altitude animals. MN•CS•IO system data demonstrate that HBB determines high‐altitude hypoxia adaptation. For validation, we generated bone marrow chimeras using CD45.1 hematopoietic cells with Pika‐HBB integrated at the Rosa26 locus. The reconstituted mice exhibited consistent hypoxic behavioral and phenotypic alterations with those observed via the MN•CS•IO system, validating the reliability and stability of HBB‐mediated high‐altitude hypoxia adaptation. Together, our findings demonstrate that HBB is a key genetic determinant of high‐altitude hypoxia adaptation, and also introduce a versatile gene‐function validation tool for evolutionary and biomedical research.

## Graphical abstract

We developed an integrative mesh‐nanorod sorting, chitosan nanoparticle delivery, and intraosseous injection (MN•CS•IO) system to investigate HBB in high‐altitude hypoxia adaptation. Transplanting Tibetan animals' HBB‐modified Lin‐ cells into mice achieved 95.59% transfection efficiency and 8.15% HBB expression. High‐altitude HBB recipients upregulated HK1, Ppp1r15a, and Rragd, activated oxygen transport and hematopoietic pathways, and showed improved behavioral performance and neuroprotection under hypoxia.

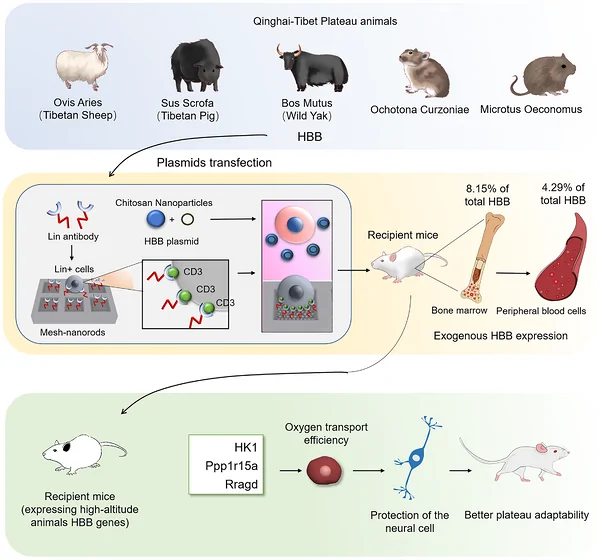

## 1 Introduction

The Qinghai‐Tibet Plateau, referred to as the “Roof of the world”, is the highest plateau on earth, with an average altitude of approximately 4300 meters [1, 2]. At high altitudes, the extreme environment, characterized by cold temperatures and reduced oxygen pressure, exerts strong selective pressures on species [3, 4]. Through long‐term natural selection, native humans and animals have developed unique physiological and genetic adaptations to high‐altitude hypoxia [5].

Identifying and verifying the genetic bases of adaptability is, therefore, crucial to functional adaptation studies in Tibetan species [6, 7, 8, 9, 10, 11, 12, 13, 14, 15]. Recent genome‐wide analyses have revealed differences between Tibetan animals and their low‐altitude counterparts, leading to the identification of potential hypoxia‐adaptive genes [16, 17, 18, 19]. HBB, a gene related to oxygen transport, has been identified as one of the candidate genes for hypoxic adaptation in humans [20, 21] and Tibetan animals, including Tibetan dogs [22, 23], sheep [24, 25, 26, 27], and pigs [28].

Although genomic studies have provided valuable insights into identifying HBB as a potential candidate gene for high‐altitude adaptation in Tibetan animals, these findings require further functional validation to fully elucidate the contribution of HBB. The critical question of whether HBB indeed leads to functional differences between high‐altitude and low‐altitude animals warrants comprehensive investigation through complementary experimental approaches.

Currently, studies on HBB function have extensively focused on the field of genetic disorders in human blood, as mutations in the HBB gene cause β‐hemoglobinopathies. These studies have primarily involved in editing the HBB gene in hematopoietic stem cells [29, 30]. Innovative techniques, such as base editing of hematopoietic stem cells, have been employed to study HBB dysfunction‐related genetic diseases [31, 32, 33, 34, 35, 36, 37].

The progress in HBB gene research has largely been driven by studies on hematopoietic stem cells. However, a critical gap remains in understanding the normal function of HBB in hypoxia adaptation, as no studies have yet investigated it using hematopoietic stem cell‐based approaches. The current research status of HBB in hypoxia adaptation is limited by the lack of an innovative research method for the rapid in vitro isolation of hematopoietic stem cells and highly efficient delivery of target genes. This limitation restricts the elucidation of the mechanisms by which HBB contributes to high‐altitude hypoxia adaptation.

Commercially and conventionally utilized experimental approaches further exacerbate these technical limitations. Transgenic mouse models suffer from long preparation periods and complicated operational procedures, whereas viral vector‐mediated gene delivery poses inherent biosafety concerns. To overcome these technical barriers, it is urgent to establish an efficient system for rapid integrated gene delivery and cell sorting tailored for hematopoietic stem cell research.

The hematopoietic stem cell from the bone marrow is a heterogeneous population of stem and progenitor cells. Among these, the lineage‐negative (Lin^−^) cells in the bone marrow comprise multipotent hematopoietic stem cells, which do not express lymphoid or myeloid lineage markers [38, 39, 40, 41].

In this context, we endeavored to investigate the HBB gene function of hypoxia adaptation through Lin^−^ cells (the antibody cocktail contains CD3, CD45R, CD11b, TER‐119, b220) and transplantation models. Herein, we developed a novel MN•CS•IO system to elucidate the function of HBB in high‐altitude hypoxia adaptation through cross‐species studies.

The schematic workflow was illustrated in Figure 1. The first step was designed to harvest Lin^−^ cells. To do this, bone marrow cells were isolated from donor mice and seeded on plates with Mesh‐nanorods (MN) substrate inserts. Next, chitosan nanoparticles (CS) encapsulated HBB plasmids from different species (sheep, pig, Yak, cattle, Pika, etc.) were delivered into mouse Lin^−^ cells. Then, upon achieving Lin^−^ cells transfected with foreign HBB plasmids cross‐species through the MN·CS system, we transplanted them into recipient mice through an intraosseous injection (IO) strategy. These above steps constituted the core MN•CS•IO technical framework of this study.

**FIGURE 1:**
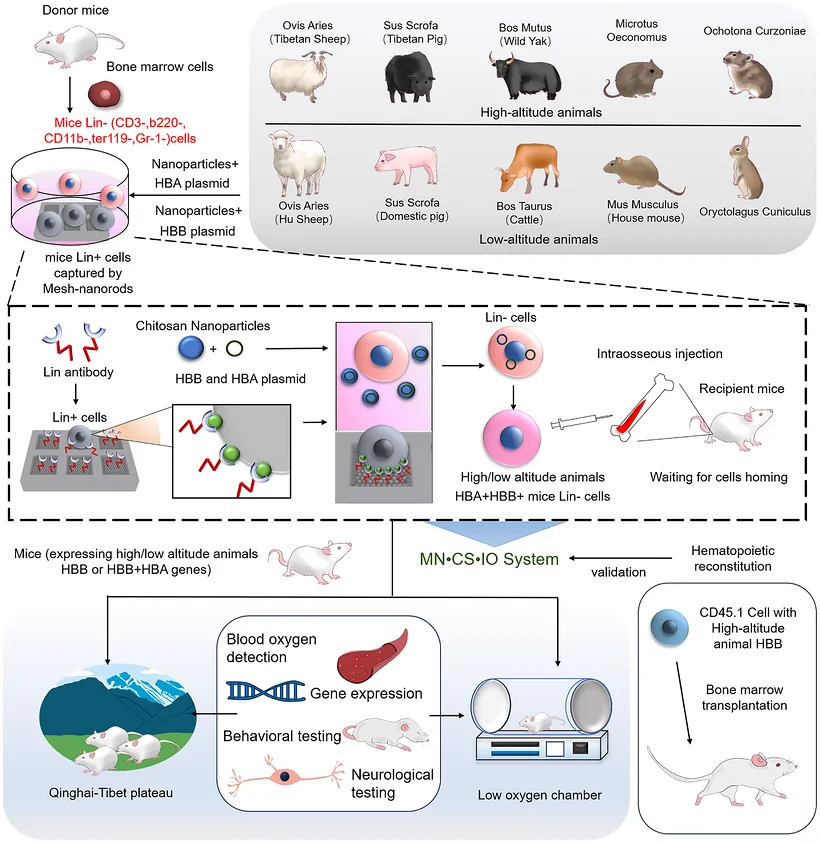
Modeling workflow for study design. The top‐left panel shows Lin^−^ cells harvested from donor mice. Bone marrow cells were first isolated from donor mice, and then seeded on Mesh‐Nanorods (MN) attached with Lin antibody to harvest Lin^+^ cells (gray, means CD3, b220, CD11b, ter119, Gr‐1. Positive for any one of the markers). Lin^−^ cells (pink, means CD3, b220, CD11b, ter119, Gr‐1 negative for all markers) from the suspension were harvested. The top right panel exhibits the foreign HBB plasmids across species from high‐altitude animals (top) and their low‐altitude counterparts (below) transfected into Lin^−^ cells by Chitosan nanoparticle (CS). Below is the detailed process of the MN·CS·IO system for recipient mouse models established. The bottom panel shows that recipient mice were evaluated both in a simulated low‐oxygen chamber and on the Qinghai‐Tibet Plateau. These evaluations aimed to investigate the physiological responses to hypoxia, including hematological indices, gene expression, behavioral testing, and neurological testing. The conventional bone marrow transplantation method in the bottom right corner was used to verify the MN·CS·IO system.

Compared with conventional bone marrow chimera‐based modeling, our MN·CS·IO system substantially reduces the time required for stable HBB establishment. It avoids lengthy donor preparation, surgical operation, and long‐term recovery of bone marrow transplantation. Its high time efficiency, simple manipulation, and safety characteristics improve its practical value and ethical suitability for animal studies, specifically enabled us to investigate the function of HBB in hypoxia adaptation between high‐altitude and low‐altitude HBB transplantation groups. Using this established system, we further evaluated comprehensive phenotypic and molecular alterations in recipient mice, including blood oxygenation indicators, gene expression profiles, behavioral patterns, and neurological functions, under both controlled low‐oxygen chamber conditions and the natural high‐altitude environment of the Qinghai‐Tibet Plateau. Conventional bone marrow transplantation mouse models were also constructed to further validate the reliability and superiority of the MN•CS•IO system in exploring HBB hypoxic adaptation functions (Figure 1).

Notably, this versatile technical platform is uniquely adaptable to cross‐species functional validation of the HBB gene under high‐altitude hypoxia. And also, it can be broadly extended to generalized gene editing research of circulatory and immune system cells, possessing great application potential in hematological and hypoxic biological research.

## 2 Results

### 2.1 MN•CS System for Mouse Lin^−^ Cell Harvest and HBB/HBA Plasmid Transfection

Before initiating our investigation into the HBB function in high‐altitude hypoxia adaptation, we developed a novel system (MN•CS) to isolate mouse hematopoietic cells and facilitate the transfection of foreign gene plasmids into these cells.

We initially tested the feasibility of this method in K562 cells for foreign HBB transfection. First, we developed an MN substrate for cell seeding, which could be directly inserted into commercial cell plates. The structure of the MN substrate was shown in Figure 2A, and its fabrication process was depicted in Figure S1A. Then, we designed a DNA plasmid transfection system utilizing CS nanoparticles encapsulated with DNA. Figure 2B illustrates the TEM analysis of the CS nanoparticle structure and the size distribution by Dynamic light scattering (DLS) analysis, with a diameter of approximately 100 nm. Afterward, we transfected CS nanoparticle‐encapsulated human HBB plasmid (harboring the tdTomato gene) to K562 cells and compared the effectiveness and safety with the conventional electroporation method. Results showed that our method was comparable to electroporation in transfection efficiency (Figure S1B–E), but superior to electroporation in maintaining cell viability (Figure S1F).

**FIGURE 2:**
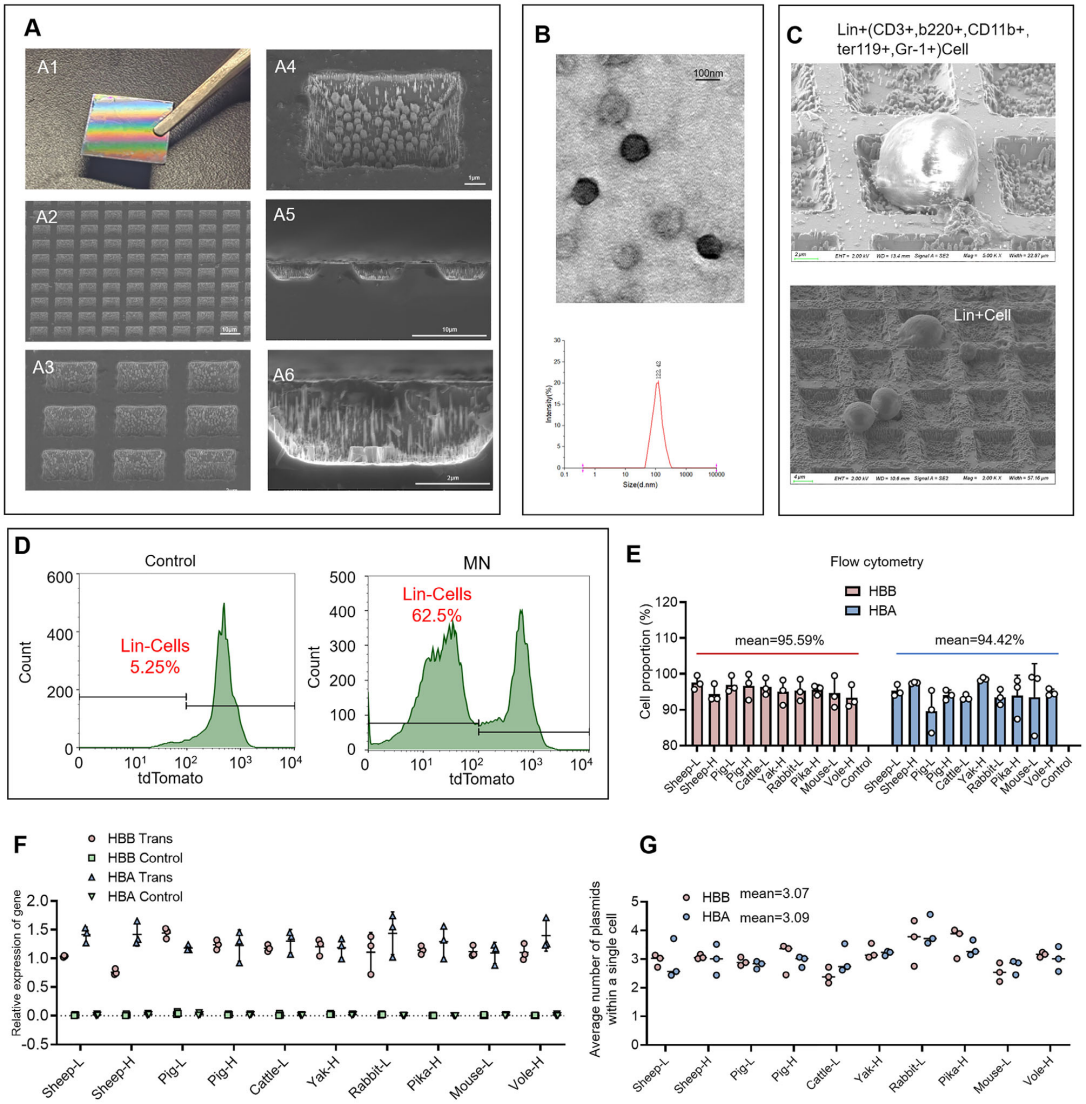
MN·CS system for K562 Cell seeding and plasmid transfection. A, Representative scanning electron microscopy images of MN. B1: A picture of MN, available for insertion into cell plates. A2‐A6: SEM images of MN (A2: scale bar, 10 µm; A3: scale bar, 3 µm; A4: scale bar, 1 µm; A5: scale bar, 10 µm and A6: scale bar, 2 µm). B, Representative TEM and DLS analysis for the characterization of CS (scale bars, 100 nm). C, SEM image of the Lin^+^ cells attached to the MN substrates. D, the proportion of Lin^−^ cells in the supernatant suspension detected by flow cytometry. E, Cell counting for tdTomato^+^ Lin^−^ cell population (red fluorescent signal), also means Lin^−^ cells successfully transfected with HBB and HBB+HBA from different animal species, as determined by flow cytometry quantitative analysis. F, G, Detection of foreign HBB and HBA in Lin^−^ cells after transfection with HBB and HBB+HBA fr)om different animal species. Relative expression of each animal's HBB/HBA genes by qPCR (F), and the number of HBB/HBA plasmids within a single cell, in Lin^−^ cells after transfection (G). E‐G, Results shown as mean ± SEM, n = 3.

Given the successful application of our MN•CS system in human HBB transfection within K562 cells, we were motivated to explore its potential for introducing various animal HBB genes into mouse Lin^−^ cells. A total of ten animals were included in this study. Five high‐altitude species (altitude ≈ 3000m) were selected: Tibetan sheep, Tibetan Yak, Tibetan pig, Tibetan Pika, and root vole. Their respective low‐altitude counterpart or relatives (altitude ≈ 30m) included Hu sheep, domestic pig, plain cattle, domestic pig, domestic rabbit, and house mouse. Plasmids targeting HBB/HBA genes for these ten animals were respectively transfected into mouse Lin^−^ cells, for further transplantation into recipient mice. Here, HBB and HBA plasmids were designed, as hemoglobin consists of two subunit types: alpha‐subunits, encoded by the HBA gene, and beta‐subunits, encoded by the HBB gene. The HBB and HBA sequence information for each animal were demonstrated in Table S1, while comparison for amino acid sequences was shown in Figure S2B,D, respectively. The predicted protein structures of HBB and HBA showed that high‐altitude species HBB display convergent evolution, characterized by reduced positive charge in the BPG‐binding pocket, elevated oxygen affinity, and enhanced structural stability, while low‐altitude species retain the ancestral phenotype of strong BPG binding and low basal oxygen affinity (Figure S2A). In addition, the HBA subunit exhibits high conservation across all species (Figure S2C). Further, Co‐immunoprecipitation (IP) analysis demonstrated that the expressed HBB can assemble with endogenous murine alpha‐globin into functional hemoglobin tetramers (Figure S2E).

First, we collected the bone marrow cells from a donor mouse and isolated Lin^−^ cell using our novel MN substrates. Figure 2C displays the harvested Lin^+^ cells attached to the MN substrates shown in the SEM image. It is noted that while the MN substrates captured Lin^+^ cells, the supernatant suspension cells harvested were Lin^−^ cells. The proportion of Lin^−^ cells in the collected supernatant suspension was 62.5%, significantly higher than 5.25% found in bone marrow cells without our MN substrate separation method (Figure 2D).

Subsequently, tdTomato‐labeled HBB/HBA plasmids of each animal were encapsulated by CS and transfected into mouse Lin^−^ cells, respectively. Then, we detected the tdTomato+ Lin^−^ cell population resulting from each transfection. Flow cytometry analysis revealed an average transfection efficiency of 95.59% for the HBB plasmid and 94.42% for the HBA plasmid (Figure 2E, Figure S3), while fluorescence microscopy showed the average efficiencies of 91.90% for HBB and 92.51% for HBA (Figure S4). Furthermore, we successfully detected the expression of each animal's HBB/HBA genes in Lin^−^ cells after transfection (Figure 2F), and quantified the number of HBB/HBA plasmids within a single cell (Figure 2G). The above results indicated that our MN•CS system could achieve a high efficiency of delivery and expression of foreign HBB/HBA genes in mouse Lin^−^ cells.

To further assess the system's functionality, we evaluated the temporal stability of foreign plasmid uptake. After transfecting Lin^−^ cells with a tdTomato‐labeled human HBB plasmid, we observed a robust and sustained tdTomato signal for up to 18 days, followed by a gradual decline with a weak signal persisting at 30 days, as shown from fluorescence microscopy images (Figure S5A) and flow cytometry analysis (Figure S5B). Consistent findings were also observed for mRNA expression of tdTomato (Figure S5C) and HBB (Figure S5D), as well as the average plasmid number within a single cell (Figure S5E). Collectively, these data suggested that our MN•CS system effectively enabled mouse Lin^−^ cell harvest, HBB/HBA plasmid transfection, and durable gene expression for approximately 15 days.

### 2.2 MN•CS•IO System: Intraosseous Injection of HBB‐transfected Donor Lin^−^ Cells Into Recipient Mice

Since the above experiments describe the feasibility of our MN•CS system in durable foreign HBB/HBA expression in vitro, we sought to perform in vivo studies for investigating into the function of HBB for high‐altitude hypoxia adaptation.

First, we attempted to transplant foreign HBB‐ (from high‐ and low‐altitude species) modified Lin^−^ cells into murine models. To do this, we first investigated the typical range of HBB‐modified Lin^−^ cells injected per recipient mouse, and found that the dose of 3 × 10^7^ Lin^−^ cells isolated from 3 donor mice for per recipient worked best (Figure S6A,B). Then, we explored the injection strategy and observed the IO injection group showed significantly higher HBB expression than the tail vein injection group (Figure S6C–E). MN•CS•IO system was therefore utilized to establish the murine models. i.e., 3 × 10^7^ Lin^−^ cells were isolated from three donor mice by MN, foreign HBB/HBA plasmid was then transfected into these cells by the CS system. Finally, these modified cells were injected into recipient mice via an intraosseous strategy (IO) (Figure 3A).

**FIGURE 3:**
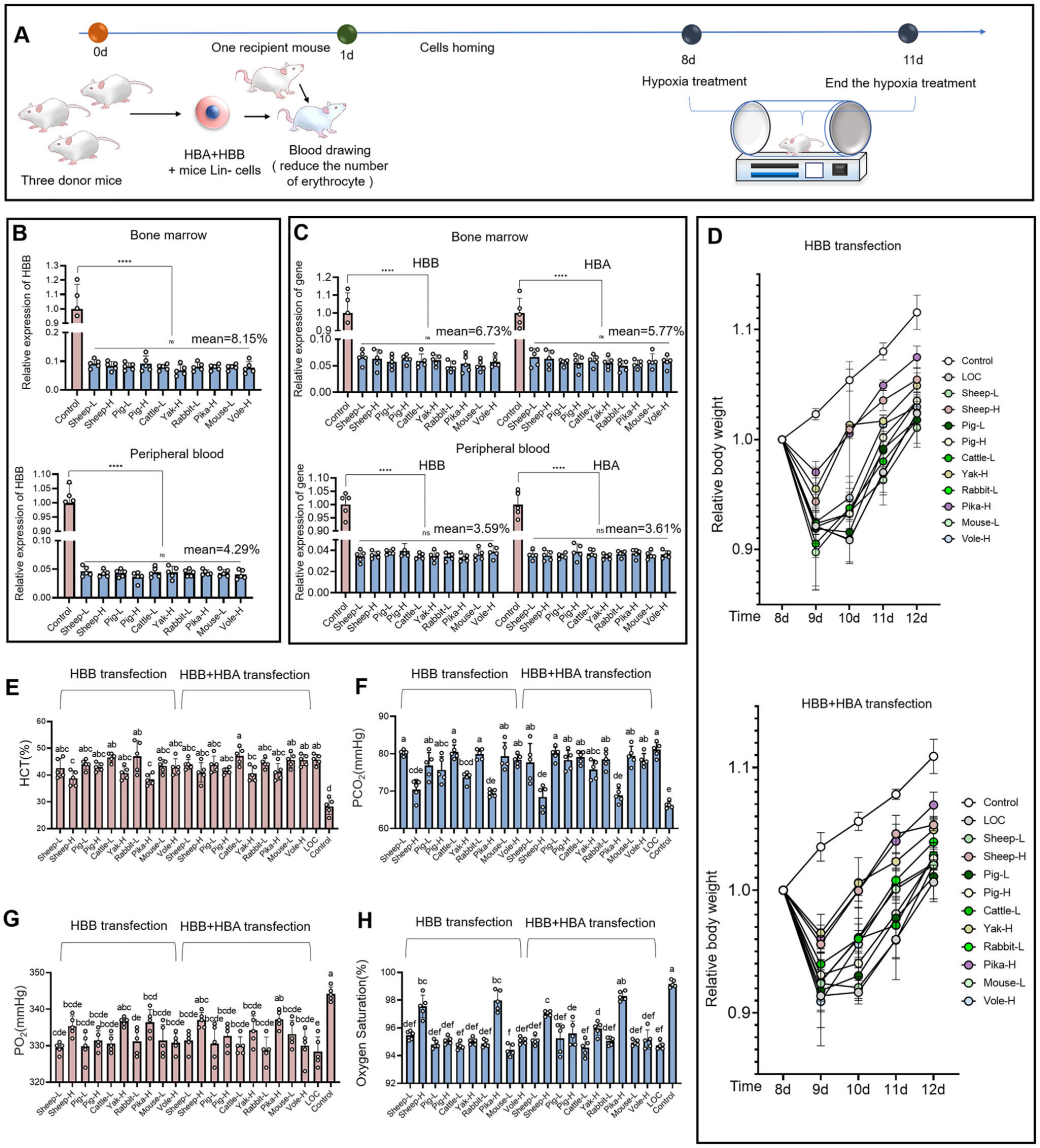
The transfection efficiency of HBB and HBB+HBA plasmids in recipient mice, and changes in body weight and blood gas parameters after hypoxia chamber treatment. A, Experimental schematic for establishment of transplantation mouse models via intraosseous injection of Lin^−^ cells transfected with foreign HBB gene, and schematic for experiment in low oxygen chamber, after establishment of transplantation mouse models via intraosseous injection of Lin^−^ cells transfected with foreign HBB/HBB+HBA gene. B, Relative expression of foreign HBB in the bone marrow cells (Top) and the peripheral blood cells (Below) of recipient mice, with transplantation of Lin^−^ cells with human HBB transfection. C, Relative expression of foreign HBB and HBA in the bone marrow cells (Top) and the peripheral blood cells (Below) of recipient mice, with transplantation of Lin^−^ cells with HBB+HBA co‐transfection. D. The changes in body weight of transplantation mice from the time they entered the hypoxia chamber until 1 day after they exited the chamber (days 8–12). HBB transplantation mice (Top) and HBB+HBA transplantation mice (Below). B‐D. Results shown as mean ± SEM, n = 5. E‐H. The following parameters were analyzed in recipient mice with transplantation of Lin^−^ cells transplanted with HBB from high‐altitude and low‐altitude animals in a low‐oxygen chamber, and un‐transfected mice under both hypoxic (LOC) and normoxic conditions (Control). The hematocrit (Hct) values (E), pCO_2_ levels (F), pO_2_ levels (G), and Oxygen saturation value (H). Results are shown as mean ± SEM, n = 5, P values were calculated using one‐way ANOVA and Tukey tests. Different lowercase letters above the bars indicate significant differences among groups (P< 0.05).

Upon the establishment of in vivo mouse transplantation models, we tried to evaluate the stability and durability of foreign plasmid transfection in those recipient mice. After transplantation, we observed durable expression of foreign human HBB expression in recipient mice at 15 days post‐injection, followed by a decline and maintained expression up to 20 days (Figure S6F). A similar pattern was observed in the average number of plasmids within a single cell (Figure S6G). Furthermore, we assessed transplantation safety by measuring immunological factors (IL‐17, TNF‐α, IFN‐γ, IL‐6) in recipient mice receiving donor Lin^−^ cells transfected with foreign animal HBB, with untransfected controls included. No inter‐group differences were observed to indicate donor‐recipient immunological incompatibility (Figure S6H). Moreover, we found the average expression of the foreign animal HBB gene was approximately 8.15% of the endogenous HBB gene expression in bone marrow cells and 4.29% in peripheral blood cells of the recipient mouse (Figure 3B). In addition, we observed our system could also achieve successful co‐transfection of HBB and HBA plasmids (Figure 3C). These results suggested a successful establishment of in vivo mouse models of foreign HBB transplantation via the above MN•CS•IO system.

Next, we headed to perform the hypoxia study. We used a low‐oxygen chamber to simulate the environment of an altitude of 5,000 meters above sea level, with the air pressure set at 55–60 kPa. At 8 days post‐transplantation of Lin^−^ cells transfected with HBB or HBB+HBA plasmids from different animals, these recipient mice and untransfected groups were housed in a low‐oxygen chamber for 3 days (Figure 3A). Untransfected mice under normoxic conditions were used as controls.

First, we compared the changes in body weight of mice from the time they entered the low‐oxygen chamber until 1 day after they exited the chamber. All mice exhibited weight loss after entering the hypoxic environment. However, Pika HBB recipient mice and Tibetan sheep (sheep‐H) HBB recipients relatively mitigate the extent of weight loss by day 9 (Figure 3D). Then, to assess the potential difference in hypoxia responses in high‐altitude HBB transplantation mice compared to their low‐altitude counterparts, we measured the blood gas parameters, including haematocrit (Hct), partial pressure of CO_2_ (pCO_2_), partial pressure of O_2_ (pO_2_), and blood oxygen saturation (SaO_2_) in the HBB transfection mice under hypoxia, un‐transfection mice under hypoxia (LOC) and normoxic condition (Control). We found that compared to normoxic controls, LOC mice exhibit significant changes in blood gas parameters, including Hct, pCO_2_, pO_2,_ and SaO_2_ (Figure 3E–H). However, transfection of HBB or HBA+HBB plasmid from high‐altitude species ameliorates these alterations under hypoxia, a function not possessed by HBB from low‐altitude plain animals. These significant changes for the reversal were observed between HBB transplantation mouse models of sheep‐H (Tibetan sheep) vs. Sheep‐L (Low‐altitude Hu sheep), Tibetan Yak vs. Low‐altitude Cattle, and Plateau pika vs. Rabbit. This finding is consistent with the difference in weight changes. In addition, we measured the hematological indicators (Table S2). What's more, we observed that results from both body weight and blood oxygen measurements revealed no significant differences between the group co‐transfected with HBB+HBA and those transfected with HBB alone, under the same treatment conditions. Therefore, we opted to use only the HBB transfection treatment in subsequent experiments.

### 2.3 Changes in Physical Fitness and Behavior in Recipient Mice Transplanted HBB From High‐/Low‐Altitude Species After Exposure to Simulated and Natural Qinghai‐Tibet Plateau Hypoxic Conditions

In the low‐oxygen chamber, we observed that mice transplanted HBB from different altitudes showed changes in body weight, so we were curious to see whether there is difference in their behavioral performance. First, we performed studies in simulated hypoxic conditions. After being housed in a low‐oxygen chamber for three days, mice were performed treadmill experiments and the Open‐field test to analyze their behavior (Figure S7A). In the treadmill test, for the number of mice falling from the treadmill, few drop‐offs were observed in the Pika‐HBB recipients (Figure S7B). In addition, in the Open‐field test, hypoxia significantly reduced the average movement speed of hypoxic mice compared to normoxic mice. However, transplantation of HBB from high‐altitude animals significantly increased average speed, time spent in the central area, and reduced freezing time in mice under hypoxic conditions. This effect was not observed in the low‐altitude HBB plasmid transplantation group (Figure S7C1–C4). It may suggest that introducing the HBB gene from high‐altitude species leads to increased active motor awareness in mice under hypoxic conditions.

Further, we were also curious to evaluate the potential effects of high‐altitude animal HBB on the behavioral performance and cognitive ability of recipient mice under hypoxic conditions. However, it is challenging to train mice in a low‐oxygen chamber, which makes it difficult to conduct training‐based behavioral experiments in the laboratory. Therefore, we performed studies under natural high‐altitude hypoxic conditions, the Qinghai‐Tibet Plateau, at an altitude of 3800 meters above sea level. Following the establishment of HBB‐transfected Lin^−^ cell transplantation mouse models via the MN•CS•IO system, mice were housed normally at the animal center of Qinghai University Affiliated Hospital for six days. Afterward, they were transported to the animal center of Henan County Mongolian and Tibetan Hospital for behavioral experiments (with the altitude of 3,800 meters) (Figure 4A). Three days later, animal behavior experiments and neurological testing were performed.

**FIGURE 4:**
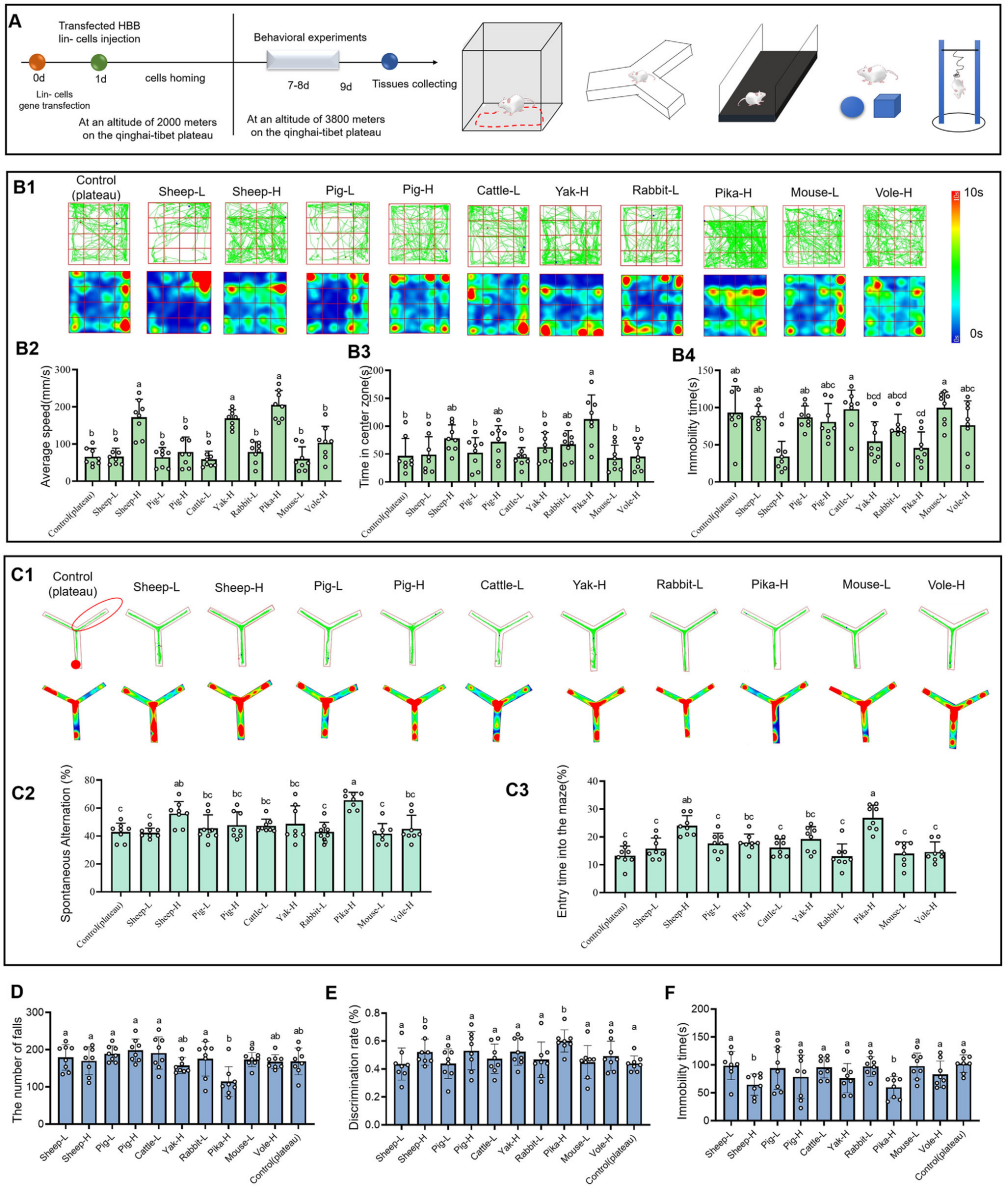
Behavioral testing of cross‐species HBB transplantation mice under Qinghai‐Tibet Plateau hypoxic environment. A. Workflow model of cross‐species HBB transplantation mice experiment on the Qinghai‐Tibet Plateau. B. Behavioral testing in foreign HBB‐transplanted mouse models in the open‐field test. B1. Representative heatmaps and trajectory maps. B2. Movement speed detection. B3. Time in center zone. B4. Immobility time. C. Behavioral testing in foreign HBB‐transplanted mouse models by Y‐Maze. C1. Representative heatmaps and trajectory maps (the red marking indicates the new arm). C2. Spontaneous alternation calculated as the actual number of alternations/ the total number of arm entries−2. C3. Entry time into the maze analyzed. D. The number of falls in the treadmill experiments of the groups. E. Discrimination rate of mice with new object recognition. F. Immobility times of mice in the tail suspension experiment. B‐F. Results are shown as mean ± SEM, n = 8; P values were calculated using one‐way ANOVA and Tukey tests. Different lowercase letters indicate significant differences among groups (P< 0.05).

First, we conducted an open‐field test and found similar results on the Qinghai‐Tibet plateau to those in the low‐oxygen chamber (Figure 4B1). Notably, Pika HBB and Tibetan sheep (sheep‐H) HBB transplantation mice exhibited significant differences in the following aspects of the open‐field test, including average speed (Figure 4B2), time in the central area (Figure 4B3), and freezing time (Figure 4B4). Subsequently, we performed a Y‐maze test (Figure 4C1) and observed that high‐altitude animal HBB transplantation mice had longer exploration periods (Figure 4C2) and higher exploration frequencies in the novel arms of Y‐maze (Figure 4C3), indicating reduced spatial reference memory impairment, compared to low‐altitude transplantation counterparts. In addition to the treadmill test, which showed similar results to those observed in the low‐oxygen chamber (Figure 4D), we performed a new object recognition experiment to assess cognitive performance and found significant differences between the high‐altitude HBB group and their low‐altitude counterparts, especially for Tibetan sheep from high‐altitude (sheep‐H) and Pika HBB transplantation groups (Figure 4E). What's more, the tail suspension test revealed low‐altitude HBB group had a longer immobility time than their high‐altitude HBB counterparts (Figure 4F).

These findings suggested that under hypoxic conditions, high‐altitude HBB recipients exhibited enhanced physical fitness and improved behavioral performance and cognitive ability, as well as reduced anxiety, when compared to low‐altitude counterparts.

### 2.4 The Mechanism Underlying the Superior High‐Altitude Hypoxia Adaptation of High‐Altitude Species HBB Transplantation Mice

Furthermore, we were keen to investigate the mechanism that underlies the difference in hypoxia response of HBB‐transfected Lin^−^ cells in recipient mice. First, we performed transcriptome sequencing in the peripheral blood and bone marrow cells of transplantation mice in the low oxygen chamber to simulate hypoxic conditions. Generally, for gene expression pattern in peripheral blood cells, hypoxic mice clustered together and were significantly separated from the normoxic controls, as can be seen from principal component analysis (PCA) (Figure 5A) and gene expression cluster analysis (Figure 5B). Moreover, under the same hypoxia condition, recipient mice transplanted with HBB from high‐altitude animals clustered together and were separated from their low‐altitude counterparts. Similar results were found in bone marrow cells (Figure 5C,D). In addition, for gene expression profiles, hypoxia in mice caused significant changes detected both in peripheral blood and bone marrow cells. GO and KEGG analyses were shown in Figure S8. Representative differentially expressed genes were demonstrated in Figure 5E (peripheral blood cells) and Figure 5F (bone marrow cells). Notably, HK1, Ppp1r1r15a, and Rragd exhibited similar differential patterns both in bone marrow and peripheral blood cells. mRNA expression detected by qPCR showed the same trend as transcriptome sequencing (Figure 5G–I for peripheral blood cells, and Figure 5J–L for bone marrow cells).

**FIGURE 5:**
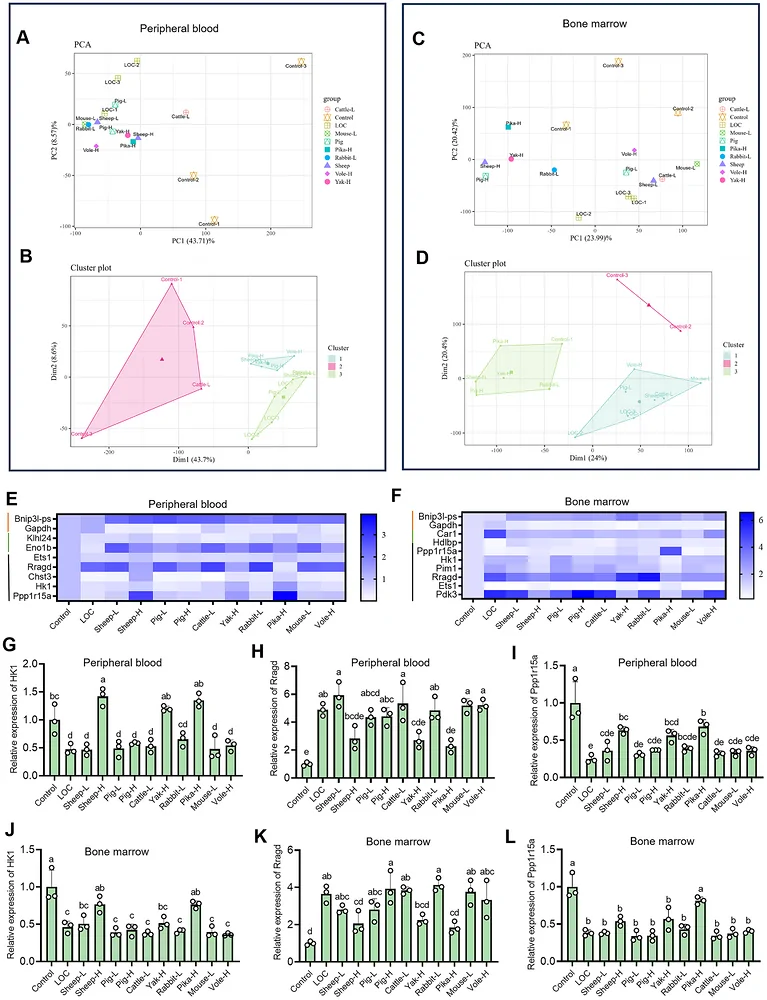
PCA analysis, clustering, and qPCR validation of differentially expressed genes from RNA sequencing in peripheral blood cells and bone marrow cells of recipient mice from different transplantation groups. A. Principal component analysis (PCA) of RNA sequencing from peripheral blood samples of different groups. Each point represents an individual sample projected onto the first two principal components (PC1 and PC2), which explain 43.71% and 8.57% of the variance, respectively. B. Cluster analysis of RNA sequencing in peripheral blood samples of different groups. Samples are grouped into three clusters, with different colors representing each cluster (Dim1 represents 43.7% and Dim2 represents 8.6%). C. Principal component analysis (PCA) of bone marrow cell samples from different groups. Each point represents an individual sample projected onto the first two principal components (PC1 and PC2), which explain 23.99% and 20.42% of the variance, respectively. D. Cluster analysis of peripheral blood samples of different groups. Samples are grouped into three clusters, with different colors representing each cluster (Dim1 represents 24% and Dim2 20.4%). E, F. Heatmap of differential expression genes in the peripheral blood cells (E) and bone marrow blood cells (F). G‐L. Relative expression of genes including HK1, Ppp1r1r15a, and Rragd in peripheral blood cells and bone marrow cells of different groups, detected by qPCR. Results are shown as mean ± SEM, n = 3; P values were calculated using one‐way ANOVA and Tukey tests. Different lowercase letters indicate significant differences among groups (P< 0.05).

Furthermore, we analyzed the sequencing data of recipient mice transplanted with HBB from different species. We were attracted by Pika‐HBB recipients. The volcano plots (Figure S9A for peripheral blood cells and Figure S9B for bone marrow cells) illustrated the differentially expressed genes between the hypoxia recipients and the untreated normoxic controls. Second, the heatmaps (Figure S9C for peripheral blood and Figure S9D for bone marrow cells) showed the comparison of differentially expressed genes between Pika and LOC groups. Interestingly, GO enrichment analysis revealed that “Oxygen transport” and “Oxygen binding” were enriched in both peripheral blood cells (Figure S9E) and bone marrow cells (Figure S9F). Additionally, in bone marrow cells, “Immune response” and “Hemoglobin complex” are also noteworthy. The KEGG pathway analysis indicates that the “Hematopoietic cell lineage” pathway is present in both peripheral blood cells (Figure S9G) and bone marrow cells (Figure S9H). Then, we performed gene knockdown/rescues assays for Rragd (Figure S10), which was identified from transcriptome analysis and downregulated in mice receiving high‐altitude HBB, to verify the HBB downstream regulation in vivo. Recipient mice housed in a low‐oxygen chamber were divided into the following groups. Rragd inhibition + Pika HBB co‐transfection mice, Rragd overexpression + Pika HBB transfection mice, Pika HBB transfection mice, and untransfected normoxia controls. Behavioral impairments were observed in the Rragd overexpression group relative to the Pika HBB group, with altered performance in open‐field, Y‐maze, and tail‐suspension tests that resembled the LOC phenotype. In contrast, Rragd inhibition yielded beneficial outcomes comparable to the Pika HBB group without significant differences (Figure S11A–D). These findings suggest that the protective effect of exogenous plateau Pika HBB acts in an Rragd‐expression‐dependent manner, and support our transcriptome‐derived hypothesis.

In addition to transcriptome analysis, we performed histological analysis of mice under natural hypoxia conditions, Qinghai‐Tibetan Plateau. Mice were divided into i) Plain control (untransfected mice housed in normoxic conditions of Plain), ii) Plateau control (untransfected mice transported to the plateau with the altitude of 3,800 meters), and iii) high/low‐altitude animals HBB transplantation mice (transported to the plateau with the altitude of 3,800 meters), the subgroups of which were established based on the transfection of HBB from different species. Results revealed that compared with normoxic Plain controls, the Plateau control mice showed endothelial cell damage and reduced pulmonary vascular density in lung tissues. However, high‐altitude animals HBB transplantation mice improved lung tissue characteristics and showed significant differences compared with their low‐altitude counterparts (Figure 6A). Moreover, compared with normoxic Plain controls, high‐altitude Plateau control mice exhibited signs of neuronal atrophy in the brain tissue, characterized by darkly stained nuclei and a disorganized arrangement of neurons, suggesting that high‐altitude hypoxia induced mice neurons injury. However, under the same conditions, high‐altitude animal HBB transplantation mice demonstrated a noticeable improvement, while their low‐altitude counterparts did not possess this significantly improve character (Figure 6B and Figure S12A).

**FIGURE 6:**
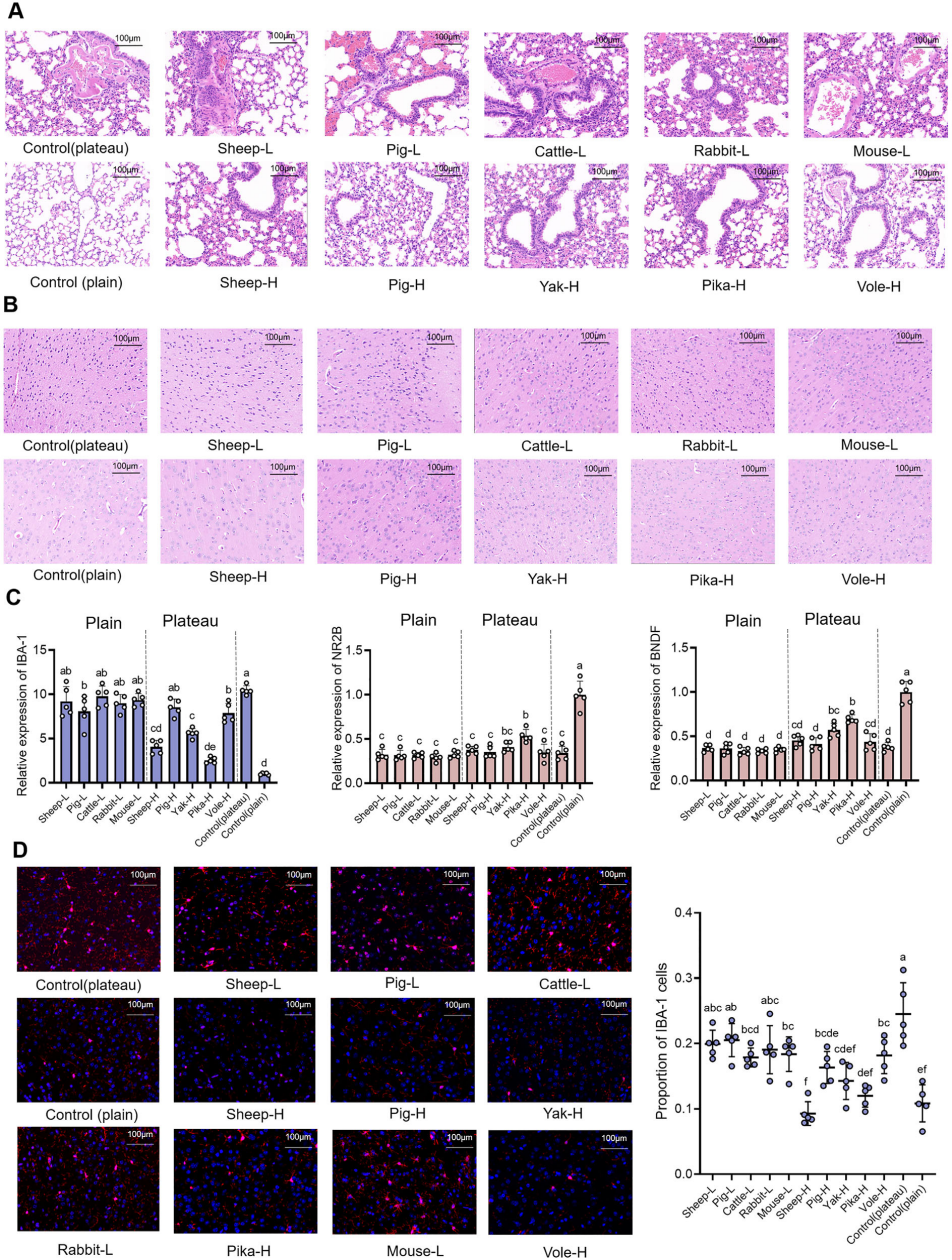
Histology analysis in cross‐species xenogeneic HBB‐transplanted mouse models on the Qinghai‐Tibet Plateau. A. HE histology analysis of the lung tissue in the cross‐species xenogeneic HBB‐transplanted mouse models on the Qinghai‐Tibet Plateau, with the altitude of 3,800 meters. Scale bars, 100 µm. B. Representative micrographs of HE‐stained sections of brain tissue. Tissues were harvested from exogenic HBB recipient mice in the natural high‐altitude of Plateau and untransfection in Plateau (Plateau control) and Lowland plain (Plain control). Scale bars, 100 µm. C. Relative expression of genes associated with the nervous system, including IBA‐1, BNDF, and NR2B. D. Immunofluorescence single labeling of IBA‐1 on paraffin sections of the mouse brain tissue (Left), and proportion of IBA‐1 cells (Right). Scale bars, 100 µm. A‐D. Control (Plateau): Mice without HBB transfection, which were transported to and housed on the Qinghai‐Tibet plateau with the altitude of 3,800 meters. Control (Plain): Mice without HBB transfection and housed in normoxic conditions of Plain. sheep‐L: recipient mice transplanted with HBB of low‐altitude sheep; sheep‐H: recipient mice transplanted with HBB of high‐altitude sheep. The same applies to other animals. All transplanted mice were transported to and housed on the Qinghai‐Tibet plateau with the altitude of 3800 meters. Results are shown as mean ± SEM, n = 5; P values were calculated using one‐way ANOVA and Tukey tests. Different lowercase letters above the bars indicate significant differences among groups (P< 0.05).

Then, we detected mRNA expression of the IBA‐1, NR2B, and BNDF genes. IBA‐1, a biomarker of microglia, is triggered to activate, proliferate, and migrate to injury sites under hypoxia [42]. NR2B and BNDF are associated with the survival and function of brain cells and neurons [43, 44]. First, we found that after being transported to the high altitude of the Qinghai‐Tibet plateau, mice exhibited significant differences in the expression of these genes (significantly higher levels for IBA‐1 and significantly lower levels of NR2B and BNDF), when compared with Plain controls. Further, we found that IBA‐1 in the high‐altitude HBB transplantation group is significantly lower than that of untransfected plateau controls, when at the same environment. While NR2B mRNA expression in the Pika‐H group exhibited significant differences compared to the untransfected plateau controls. BNDF mRNA expression in Yak‐H and Pika‐H groups showed significant differences compared to the untransfected Plateau controls (Figure 6C).

Further, we focused on the biomarker of microglia, IBA‐1. Immunofluorescence analysis of the mouse brain tissue revealed increased microglial activation in the Plateau untransfected mice, when compared with Plain control, suggesting increased neuronal injury sites of mice under hypoxic conditions. However, in mice transplanted with HBB from high‐altitude species, the activation of IBA‐1 was inhibited. We speculated that HBB from high‐altitude animals may suppress the neurological injury induced by hypoxia. Whereas IBA‐1 in their low‐altitude counterparts remained activated. This is consistent with the above qPCR results. IBA‐1 cell proportion results also verified this finding. We found that IBA‐1 cells proportion is significantly higher in Plateau untransfected mice than that of Plain controls, while transplantation of HBB from high‐altitude significantly reversed this phenomenon (Figure 6D and Figure S12B). Combined with histology analysis and mRNA expression, it suggested that the HBB of high‐altitude animals may exert a protective effect on neurological damage caused by hypoxia.

Collectively, high‐altitude HBB down regulates Rragd, and activates oxygen transport and hematopoietic lineage pathways under hypoxia. Lung and brain histology confirms that HBB alleviates hypoxic injury by reducing microglial IBA‐1 and restoring NR2B and BDNF. However, these protective effects are not absent in mice with low‐altitude HBB.

### 2.5 The Verification of our MN•CS•IO System by Traditional Bone Marrow Chimeric Mice Model

Further, to track and verify the contribution of HBB genes to hypoxic adaptation used by our MN•CS•IO System, we established CD45.1 haematopoietic cell bone marrow chimeric mice. First, CD45.1 cells were sorted from a donor SJL/OrlcoCr mouse. Next, we introduced the Pika‐HBB gene and the GFP gene (an eGFP‐HBB cassette) into the Rosa26 locus site via homologous recombination in CD45.1 cells. Then, we sorted Pika HBB∈CD45.1 cells with GFP fluorescence using flow cytometry, and intravenously injected them into CD45.2‐expressing C57BL/6J recipient mice that had been subjected to myeloablation. At 12 weeks post‐transplant, we collected bone marrow tissues to verify the success of the transplantation (Figure 7A). Flow cytometry analysis of the CD45.1/CD45.2 ratio showed successful bone marrow chimeras model establishment (Figure S13A), although mice after myeloablation showed poorer physical condition than normal mice, as can be seen from the results of the treadmill test (Figure S11B). On the other hand, electrophoresis and DNA sequencing confirmed the correct integration of HBB gene editing at ROSA 26 sites (Figure S13C–E).

**FIGURE 7:**
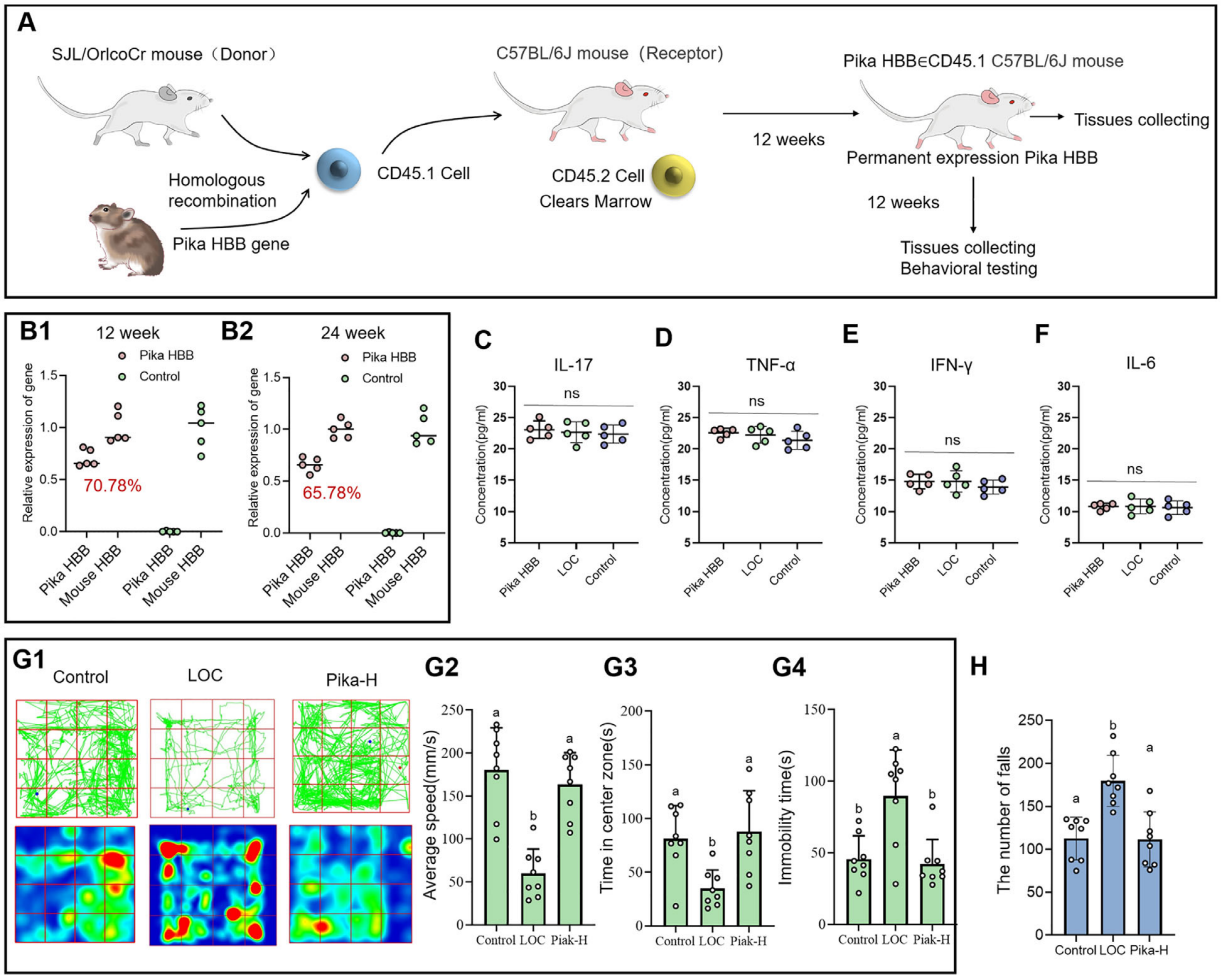
HBB function investigation and MN•CS•IO System verification in traditional Bone marrow chimeras mice. A. Schematic illustration of Pika HBB∈CD45.1 bone marrow chimeras mice model establishment. B. Relative expression of foreign HBB in the peripheral blood cells of recipient mice, with transplantation of Pika HBB∈CD45.1. C. ELISA detection of IL‐17, TNF‐α, IFN‐γ, and IL‐6 inflammatory factors (C1‐C4). Pika HBB means myeloablation mice + transplantation of Pika HBB∈CD45.1; after 24weeks, mice were subjected to myeloablation and housed in a low‐oxygen chamber for 4 days. LOC means myeloablation mice without transplantation and housed in a low‐oxygen chamber for 4 days. Control means myeloablation mice without transplantation and housed in normoxic conditions. The same below. D1. Representative heatmaps and trajectory maps of mice in the open field. D2. Movement speed detection. D3. Time in the center zone. G4. Immobility time. E. The number of falls in the treadmill experiments of the groups. B‐C. Results are shown as mean ± SEM, n = 5; P values were calculated using one‐way ANOVA. Different lowercase letters above the bars indicate significant differences among groups (P< 0.05). D, E. Results are shown as mean ± SEM, n = 8; P values were calculated using one‐way ANOVA. Different lowercase letters above the bars indicate significant differences among groups (P< 0.05).

HBB expression by Real‐time PCR showed the average engraftment percentage (70.78%) after 12 weeks of transplantation and (65.78%) after 24 weeks of transplantation (Figure 7B). The bone marrow chimeric mice, when placed in a low‐oxygen chamber for 4 days, showed no significant differences in inflammatory factors, including IL‐17, TNF‐α, IFN‐γ, and IL‐6, compared with the normoxic group (Figure 7C). The open‐field test showed that Pika HBB transplantation mice exhibited significant differences in trajectory (Figure 7D1), average speed (Figure 7D2), time in the central area (Figure 7D3), and freezing time (Figure 7D4), when compared with the untransplantation group. The treadmill test showed that bone marrow chimeric mice exhibited a higher number of falls compared to control mice, indicating potential deficits in motor coordination and endurance following bone marrow transplantation (Figure 7E). These results in bone marrow chimeric mice are consistent with those observed in mice using our MN•CS•IO System. The traditional method thus verified the feasibility of our approach and confirmed the findings that high‐altitude HBB recipients exhibited enhanced oxygen transport, improved behavioral performance, and neuroprotection against hypoxia‐induced damage, and showed better plateau adaptability than low‐altitude species HBB recipients.

## 3 Discussion

Hemoglobin, consisting of HBB and HBA, is considered as a crucial candidate gene for the high‐altitude adaptation of Tibetan animals. However, the function of HBB in high‐altitude hypoxia adaptation remains largely elusive, primarily due to the lack of innovative research methods. Here, we demonstrated a novel MN•CS•IO system for cross‐species studies to elucidate the function of HBB in high‐altitude hypoxia adaptation.

First, in terms of methodology: we pioneered a new method of investigating the function of HBB in high‐altitude hypoxia adaptation by utilizing hematopoietic cells. Although extensive studies have been conducted in hematopoietic cells for human HBB dysfunction research, such as genetic disorders like thalassemia and sickle cell anemia, no reports have been published specifically on HBB hypoxia adaptation through a hematopoietic cell‐based approach. Our innovative MN•CS•IO system approach offers a novel perspective for studying HBB for hypoxia adaptation, particularly in understanding its physiological functions and mechanisms. In this study, we successfully developed an in vitro MN•CS system, with the highly efficiency of 95.59% for HBB and 94.42% for HBA. Upon the cell harvest and gene delivery process, we developed a transplantation protocol to introduce the hematopoietic cells harboring foreign HBB into recipient mice. Our MN•CS•IO system paves the way for gene function research within hematopoietic cells. When compared to the conventional method of utilizing bone marrow chimeras for this purpose, our approach offers significant advantages, particularly in terms of time efficiency. Our procedure reduced the timeline for establishing the HBB model. While traditional bone marrow transplantation often requires at least 12 weeks to complete due to the complex steps involved [45, 46], including donor preparation, transplantation surgery, and prolonged postoperative recovery, our approach can achieve stable HBB establishment within a much shorter time. Furthermore, our method is more convenient to perform and is nearly non‐invasive to the animals, which are additional benefits that enhance its application and ethical acceptability in research.

Second, for the HBB function investigation: In terms of foreign gene expression, our innovative MN•CS•IO system enables the expression of the foreign HBB gene at levels comparable to the robust expression of the endogenous HBB gene in recipient mice. Also, it induces phenotypic differences in HBB‐transfected Lin^−^ cell transplantation mice under high‐altitude hypoxia conditions. As expected, following the transplantation of Lin^−^ cells harboring the foreign HBB, we detected a modest approximately 3.59‐8.15% increase in HBB/HBA production, relative to the mice's own inherent HBB level, in recipient mice. Further, we found that high‐altitude Tibetan species HBB transplantation mice exhibited a mitigation of alterations induced by hypoxia, including blood parameters, oxygen transport‐related gene expressions, and behavior, and may protect the neural system from damage caused by hypoxia, which were not possessed by their low‐altitude counterparts. It is evident that these changes in the recipient mice are attributed to the function played by unique donor HBB from high‐altitude animals, rather than simply to an increase in total hemoglobin caused by foreign HBB transfection. It is also an important reason for the adaptability of high‐altitude animals to their environments.

Moreover, our study represents a breakthrough in the current field of study on HBB function in high‐altitude hypoxia adaptation. As a complement to traditional studies based on genome sequencing results, and differing from it in approach, our study validated the HBB function of high‐altitude adaptive responses and directly confirmed its adaptive function to the high‐altitude environment. It provides evidence that animals inhabiting the Tibetan Plateau have developed unique adaptations, potentially mediated by HBB, to respond to the challenge of the high‐altitude environment. And also, it could help bridge the current research gap and provide a more profound understanding the function of HBB in high‐altitude adaptation.

Last but not least, the potential significance of our methodological investigation extends beyond the study of the HBB function for hypoxia adaptation. It also offers innovative approaches and methodologies for other genes' function research in both wild and domestic animals, which are typically not suitable or have challenges in being used as model animals, but still hold significant value in genetic research.

## 4 Experimental Section/Methods

### 4.1 Animals

Male C57BL/6 mice (4–6 weeks old) and SJL/JorllcoCrl mice, weighing 22 ± 2 g, were purchased from Charles River Laboratories (China). For the simulated oxygen chamber experiment, mice were housed in the animal facility of the Center for Stem Cell and Translational Medicine, School of Life Sciences and Medical Engineering, Anhui University (Hefei, Anhui, China). For the Qinghai‐Tibet Plateau experiment, mice were housed in the animal center of the Medical Department of Qinghai University for 6 days (Xining, Qinghai, China). Afterward, they were transported to the animal center of Mongolian and Tibetan Hospital located in Henan County, Huangnan Tibetan Autonomous Prefecture, Qinghai, China, for further experiments.

All the housing environment was regulated with a 12 h light/dark cycle. The ambient temperature was maintained at 24 ± 2°C, and the humidity was kept at 50 ± 10%. Commercial mouse diet and bottled double‐distilled water were available and libitum. During the adaptation of 6 days, mice were randomly housed in different groups in cages.

All animal studies were approved by the Institutional Animal Care and Use Committee of Anhui University (IACUC(AHU)‐2024‐016) and Animal Ethics Committee of Anhui Academy of Agriculture Sciences (AAAS2022‐10).

### 4.2 Fabrication of Mesh‐Nanorod (MN) Structure

First, positive photoresist BCI 3511 was spin‐coated on a SiO2/Si (100) (SiO2: 100 nm in thickness) wafer at 2500 rpm. The substrate was then exposed to a mercury‐xenon lamp (3500 mW/cm^2^, 365 nm, Hamamatsu Corporation, JP) for 30 s. Immediately following exposure, the photoresist was developed for 30 s to obtain a well‐patterned mesh structure. Subsequently, the SiO2 layer was selectively removed through reactive ion etching (RIE), employing CF4 and CHF3 gases. The substrate was then immersed in a 70°C KOH solution to fabricate a 2 µm‐deep inverted square frustum structure.

Next, 200 µL of a 500 nm‐diameter polystyrene nanosphere (PS nanosphere) colloid was centrifuged to remove the supernatant. Absolute alcohol (400 µL was added to the precipitate and sonicated to obtain a homogeneous colloid. The substrate was positioned in a water‐filled Petri dish with an inclined coverslip along its edge. The PS nanosphere colloid was slowly dropped along the coverslip until a closely packed nanosphere layer with an iridescent color formed on the water surface. A pipette tip was inserted below the water surface to gently remove the water, allowing the nanospheres to settle onto the substrate. The substrate was then placed in a vacuum drying oven for 4 h to eliminate residual moisture, followed by a 10 min bake at 90°C to secure the nanospheres in place. After that, PS nanospheres on the mesh were glued down with Scotch tape.

Finally, the substrate underwent sequential oxygen plasma treatment (500 W, 5s) and dry etching with SF6 (30 sccm), C4F8 (60 sccm) gases in an inductively coupled plasma (ICP) etching machine (HAASRODE‐E200A, Leuven) to create the mesh‐nanorod structure. The nanospheres and SiO2 mesh were removed by immersion in toluene and hydrofluoric acid, respectively. For SEM analysis, a thin layer of gold was sputtered onto its surface, preparing it for observation with SEM (JEOL JSM‐6060LV).

### 4.3 Transplantation Mouse Model Establishment

#### 4.3.1 Method 1: Intraosseous Injection (IO) Lin^−^ Cells

First, the mouse for transplantation was anesthetized. After confirming the mouse was successfully anesthetized, the mouse was placed on a clean white board of the bench, with limbs fixed by tape. Prior to IO injection, the mouse was subjected to blood collection by heart puncture to reduce the impact of the mouse's own hemoglobin on the experiment. After collecting 100 µL of blood, it was performed for the following injection. The tibia of the mouse was found and surface‐sterilized with 75% ethanol. As prepared (groups were shown in Table S5) HBB transfected mice Lin^−^ cells resuspended with 100 µL PBS were filled in 1 mL syringe, which was slowly rotated and inserted into the bone marrow cavity of the posterior tibia for each injection. The number of cells for injection was shown in Table S5. In the exploration of different cell numbers for transplantation, post‐transfected Lin^−^ cells at a concentration of 3×10^7^ were selected for the subsequent transplantation and compared with the following Tail vein injection method.

#### 4.3.2 Method 2: Tail Vein Injection

Male mice weighing 20 g were selected for tail vein injection. A 50 mL homemade plastic restrainer with drilled holes to secure the mouse was utilized to fix conscious mice. The tail of the recipient mouse was placed in the right position in the hole. To facilitate tail vein injection, the tail was immersed in a 39°C water bath for 2 min, to induce tail congestion and enhance vein visibility. The injection site was then sterilized with an alcohol‐soaked cotton ball. Subsequently, 3×10^7^ Lin^−^ cells with respective HBB/HBA transfection were resuspended in 100 µL of PBS and loaded into a 1 mL syringe, ensuring that all air was expelled from the syringe barrel. The tail was positioned horizontally, and the needle was inserted smoothly into the vein at a slight angle. The cell suspension was then injected slowly and steadily to ensure uniform delivery.

### 4.4 Exogenic Animals HBB Transplantation Mice Models Establishment

For the HBB and HBB+HBA transfected Lin^−^ cells injection: A total of 60 recipient mice were divided into 20 group to receive exogenous HBB/HBA plasmids: i) five high‐altitude animal HBB plasmids, ii) five low‐land HBB plasmids, iii) five high‐altitude animal HBB+HBA plasmids, and iv) five high‐altitude animal HBB+HBA plasmids (3 mice in each group). Lin^−^ cells transfected with the respective HBB (at a density of 3 × 10^7^ cells/mL) were resuspended in 100 µL of PBS and loaded into a 1 mL syringe. The syringe was then slowly rotated and inserted to the bone marrow cavity of the mouse posterior tibia for each injection. A similar method for HBB+HBA plasmid transplantation.

### 4.5 Low‐Oxygen Chamber Experiments for Blood Indices Evaluation and Transcriptome Analysis

After transplantation, the 60 recipient mice were first housed in normoxia in standard holding conditions for 7 days. They were then transferred to a low‐oxygen chamber for 3 days (55‐60 kPa, simulating ∼ 5000 m) in low‐pressure oxygen chambers (ProOX‐810, Tawang, China).

In addition, a total of 6 mice that did not receive transplantation were divided into two groups: LOC and control, with 3 mice in each group. The LOC group in different cages was housed together with the 60 recipient mice at the same conditions, while the control group was kept under normoxic conditions until the end of the experiment. At the end of the low oxygen experiment, peripheral blood and bone marrow cells were obtained from mice in all groups.

### 4.6 Bone Marrow Cell Collection

Methods for bone marrow cell collection in all recipient mice were the same as that of above demonstrated in donor mice (Lin^−^ cells collection/ Donor bone marrow cells isolation)

### 4.7 Peripheral Blood Cell Collection

For peripheral blood cell collection: All mice were anesthetized and positioned on their side with the head pointing down. Whiskers were trimmed to prevent blood contamination. The skin around the eye was gently pulled away to expose the eyeball. A pipette tip was inserted into the corner of the eye socket beneath the eyeball at a 45‐degree angle toward the center of the socket. Gentle downward pressure was applied and released until the vein was ruptured and blood was observed entering the pipette.

The collected blood was placed into a tube containing EDTA and mixed up and down. After finishing blood collection, samples were analyzed using an automated blood gas analyzer (ProCyte DX Hematology Analyzer). For blood physiological indices analysis, blood samples were measured through the Catalyst One Analyzer (IDEXX Laboratories, Inc).

In addition, the harvested peripheral blood samples and the bone marrow cells from recipient mice were used to perform further transcriptome analysis.

### 4.8 Transcriptome Analysis

RNA samples were isolated from the bone marrow cells and peripheral blood cells by TRIzol according to the manufacturers’ instructions. Degradation and contamination of RNA was monitored on 1% agarose gels. RNA concentration was assessed by the Qubit RNA Assay Kit in a Qubit 2.0 Fluorometer (Life Technologies, CA, USA). The integrity of RNA was measured using the RNA Nano 6000 Assay Kit of the Bioanalyzer 2100 system (Agilent Technologies, CA, USA). RNA samples were sent to Wuhan BandOmics Biotechnology Co., Ltd (China), for transcriptome sequencing. For each sample, 1 µg RNA was used as input material for the RNA sample preparations. Sequencing libraries were generated using the TruSeq RNA Library Preparation Kit (Illumina, USA), and library quality was assessed on the Agilent Bioanalyzer 2100 system (Agilent Technologies, USA). Following clustering of the index‐coded samples on a cBot Cluster Generation System using TruSeq PE Cluster Kit v3‐cBot‐HS (Illumina), the library preparations were sequenced on an Illumina NovaSeq platform. The sequencing results were verified by qPCR (Methods were shown in the supporting materials). The primers listed were shown in Table S6.

To evaluate the gene expression differences among samples, Principal Component Analysis (PCA) was performed based on the gene expression levels of each sample. For differential expression analysis, DESeq2 was utilized (28). Genes were considered to be differentially expressed if they exhibited a Fold Change ≥2 and an adjusted p‐ value (FDR) ≤0.05. Additionally, GO enrichment and KEGG pathway analyses were conducted using the Bioinfo Intelligent Cloud (BIC) platform available at:https://www.bic.ac.cn/BIC/#/analysis?page = b%27MTI%3D%27&tool_type = tool

### 4.9 Low Oxygen Chamber for Behavior Testing Experiment

For the behavior testing experiment, Lin^−^ cells transfected with different animal HBB were transplanted to recipient mice. A total of 30 recipient mice were divided into 10 groups to receive exogenous HBB plasmids: i) five high‐altitude animal HBB plasmids, ii) five low‐land HBB plasmids, with 3 mice in each group. Lin^−^ cells transfected with HBB (at the density of 3×10^7^ cells/mL) were resuspended in 100 µL of PBS and loaded into a 1 mL syringe. The syringe was then slowly rotated and inserted to the bone marrow cavity of the mouse posterior tibia for each injection. After transplantation, the 30 recipient mice were first housed in normoxia in standard holding conditions for 7 days. They were then transferred to a low‐oxygen chamber for 3 days (55–60 kPa, simulating ∼5000 m) in low‐pressure oxygen chambers (ProOX‐810, Tawang, China). In addition, a total of 6 mice that did not receive transplantation were divided into two groups: LOC and control, with 3 mice in each group. The LOC group was housed together with the30 recipient mice at the same conditions, while the control group was kept under normoxic conditions until the end of the experiment.

### 4.10 Behavior Testing

#### 4.10.1 The Qinghai‐Tibet Plateau Experiment

##### 4.10.1.1 Transplantation

Lin^−^ cell transplantation was performed at the Medical Department of Qinghai University. 3 × 10^7^ Lin^−^ cells with the respective HBB plasmid transfected were suspended in 100 µL PBS, followed by filling the mixture into a 1 mL syringe. After sanitization of the mouse tibia with an alcohol swab, the needle was carefully and slowly rotated at the distal end of the tibia, near the femur, to facilitate the injection. A total of 30 mice received transplantation of HBB‐transfected Lin^−^ cells from ten different species at the Medical Department of Qinghai University, including five high‐altitude Tibetan animals HBB transplantation and five respective low‐altitude HBB transplantation groups, with 3 mice in each group. In addition, three mice without transplantation were designated as un‐transfection groups. After 7 days, these 33 mice were transported to the Mongolian Tibetan Hospital in Henan County, at an altitude of 3800m for behavioral testing experiments and sample collection for further histology. For behavioral testing, methods are shown in the supporting materials. For histology analysis, we added mice without HBB transfection and housed in Plain as the Plain control (n = 3). A total of 36 mice were divided into Plain control, Plateau control (un‐transfection mice), and HBB transfection mice.

##### 4.10.1.2 Histology

Brain and lung tissues of the HBB transfection group, un‐transfected Plateau control, and Plain control were collected for HE staining and IBA‐1 immunofluorescence.

##### 4.10.1.3 HE Staining

The brain and lung tissues from mice were fixed in a 4% paraformaldehyde solution for over 24 h at room temperature. The fixed tissues were then dehydrated as the following process: 75% ethanol for 4 h, 85% ethanol for 2 h, 95% ethanol for 1 h, 100% ethanol for 30 min (twice), Benzyl alcohol for 10 min, xylene for 30 min (twice). Then, samples were embedded and processed into wax blocks. These blocks were sectioned (4 µm) (Leica) and stored at room temperature for subsequent processing. The paraffin sections of the prepared samples were dewaxed and rehydrated by a series of solutions: xylene for 20 min (twice), 100% ethanol for 10 min (twice), 95% ethanol for 5 min (twice), 80% ethanol for 5 min, and 70% ethanol for 5min and ddH_2_O. They were then stained with hematoxylin for 3 min, followed by eosin staining for 20 min. After removing the excess stain, the sections were dehydrated and sealed. Images were collected and analyzed with a Nikon DS‐U3 camera through a microscope (Nikon Eclipse Ci‐L).

##### 4.10.1.4 IBA‐1 Immunofluorescence

The paraffin‐embedded brain slides were immersed in xylene for 15 min, then repeated for another 15 min. Then, the slides were gradually rehydrated through the following ethanol solutions: 100% ethanol for 5 min, then repeated with 100% ethanol for another 5 min, 85% ethanol for 5 min, 75% ethanol for 5 min, and finally rinsed with distilled water. After that, tissue sections were placed in a pressure cooker filled with citrate buffer (pH 6.0). Timing was initiated upon the appearance of steam and maintained for 2 min. Subsequently, the pressure cooker was cooled at room temperature. Then, the slides were washed with PBS (pH 7.4) three times. Sections were then placed in a 3% H2O2 and incubated at room temperature in the dark for 25 min to block endogenous peroxidase activity. The slides were then transferred into PBS (pH 7.4) for three washes. Subsequently, the slides were incubated in the primary antibody IBA‐1 labeled (cloneJM36‐62#et1705‐78 1:500) solution overnight at 4°C in a humidified chamber. After washing with PBS three times, the slides were incubated to the secondary antibody (Anti‐Rabbit IgG(H+L) antibody, Peroxidase‐labeled (1:500)) (conjugated with HRP) at room temperature for 60 min in the dark. After washing the slides with PBS three times, 5 µM TSA was added and incubated for 20 minu at room temperature. Following washing with PBS, 10 µL of DAPI (1 µg/mL) was applied to the slides and incubated for 10 min in the dark to stain the nuclei. Upon washing with PBS and removing excess liquid, the slides were applied to an anti‐fade mounting medium. Finally, the slides were observed under a fluorescence microscope (NIKONE CLIPSE Ti2‐U) to visualize the specific staining. LAS X Software was used for image quantitative analysis.

#### 4.10.2 Bone Marrow Chimeras

Conditioning recipient Mice with Busulfan: Before bone marrow transplantation, recipient mice require to be conditioned to deplete endogenous stem and progenitor cells and facilitate donor stem cell engraftment. For the experimental group of recipient mice (C57BL/6J mice), 10 mg/mL busulfan (MCE, #HY‐B0245) dissolved in 10% DMSO was administered intraperitoneally at 20 mg/kg/d for two days. For the control group, mice were treated with 10% DMSO. Then, blood samples were collected from the tail tip of recipient mice to verify the success of marrow depletion. The white blood cell (WBC) count in blood samples was analyzed using a Matenu blood analyzer (MTN‐31). Bone marrow depletion was considered essentially complete when the count of WBC< 1 × 10^9^/L.

##### 4.10.2.1 Donor Bone Marrow Cells Preparation

To collect bone marrow cells from donor mice, SJL/OrlcoCr donor mice (CD45.1) were sacrificed by cervical dislocation without anesthesia to prevent chemical contamination of the cells. The hind limbs were removed using forceps and sharp scissors and placed in a sterile Petri dish containing ice‐cold PBS. Then, bone marrow cells were flushed out of the bone cavities using a 1 mL syringe with ice‐cold PBS. The collected cells were treated with erythrocyte lysing solution and washed by resuspending in ice‐cold PBS. CD45 cells were isolated by a magnetic‐based sorting method (RWD Life Science Co Ltd., China, K1304‐10). In brief, a 100 µL aliquot of single‐cell suspension (1 × 10^7^ cells) was incubated with 5 µL biotinylated anti‐mouse CD45 antibody for 10 min on ice. Cells were washed with 1 mL ice‐cold buffer, pelleted at 500 × g for 5 min, and the supernatant was discarded. The pellet was resuspended in buffer, mixed with 5 µL Streptavidin MicroBeads, and incubated for a further 15 min on ice. After two additional washes, the suspension was applied to a LarSep column positioned in a magnetic field that had been pre‐equilibrated with buffer. Unlabelled (CD45^−^) cells were collected in the flow‐through, and the column was washed three times with buffer. The column was then removed from the magnet, buffer was added, and magnetically retained CD45^+^ cells were eluted by firmly pushing the plunger through the column.

##### 4.10.2.2 HBB/GFP Plasmid Construction

First, using the laboratory's in‐house HBB plasmid from mice and rabbits as a template, primers were designed with an Agel restriction site upstream of the sequence and a BstBI restriction site downstream of the sequence. The target sequence was amplified via PCR. Upon recovery of the PCR products, double digestion was performed with the laboratory's in‐house Human HBB‐GFP plasmid. The digested fragments from both reactions were recovered and subsequently ligated with T4 ligase. The ligation mixture was transformed, and a single colony was selected for sequencing verification of the successful construction of the HBB/GFP plasmid.

##### 4.10.2.3 In Vitro Delivery of Cas9/sgRNA and HBB/GFP Plasmids for Knock‐in of HBB/GFP Gene into CD45.1 Cells by Electroporation

Transfection by electroporation was conducted as follows. Cells were neutralized with fresh cell culture medium, centrifuged at 4°C (1000×g, 3 min), and resuspended in 100 µL buffer. Plasmid was added, transferred to a cuvette, and electroporated (900 V, 30 ms, 1 pulse). After electroporation, cells were resuspended in 1 mL pre‐warmed medium, seeded in 6‐well plates, and cultured at 37°C, 5% CO_2_. After transfection, the fluorescence intensity of cells was monitored using a fluorescence microscope (Leica, Germany) every 24 h. The transiently transfected cells were retained until the transient fluorescence had significantly diminished, at which point they were harvested for the subsequent experimental steps.

##### 4.10.2.4 Cell Transplantation into Recipient Mice

The prepared donor bone marrow cells (0.2 mL) were injected into the tail vein of each recipient mouse using an insulin syringe. The recipient mice were homozygous wild‐type C57BL/6J mice (CD45.2) with clear marrow. After injection, the recipient mice were rested for 3 weeks. To prevent infection, they were fed Enrofloxacin‐Water (take 50 mg of Enrofloxacin (MCE, #HY‐B0502) and dissolve in 1L clean water) for 2 weeks.

##### 4.10.2.5 Flow Cytometry Sorting

Peripheral blood tests were performed at 4, 6, 8, 12, and 16 weeks after transplantation, with blood collected from the tail vein and red blood cells lysed, then cell suspension was collected to the centrifuge tube. FITC Mouse Anti‐Mouse CD45.1 (BD Pharmingen, #561871) and PE‐Cy7 Mouse Anti‐Mouse CD45.2 (BD Pharmingen, #560696) were then added to the cell suspension. After incubated for 15 min, it was centrifuged to remove the supernatant. After removing the supernatant, the cell pellet was resuspended with 1 mL of PBS to adjust the concentration to 1×10^6^ cells/mL. After repeating the centrifugation, cells were resuspended in 500 µL of serum‐containing PBS at 1×10^6^ cells, and then transferred to a flow cytometer (BD‐FACSCalibur) to detect the FITC and PE‐A signals. Data analysis was conducted using FlowJo software to determine the proportion of CD45.1+ and CD45.2+ transplanted cells in recipient mice.

##### 4.10.2.6 ELISA

Mouse inflammatory cytokine detection was performed by using the commercial ELISA kit (Jonlnbio, Shanghai, China) (IL‐17: #JLW20251, TNF‐α: #JLW10484, INF‐γ: #JLW10967, LI‐6: #JLW20268). In brief, the plate was coated with antigen and then blocked with 5% BSA. After incubation at 37°C for 1 h, the plate was washed with PBS. The primary antibody was added and incubated, then the secondary antibody. The substrate was added, and the reaction was allowed to proceed.

### 4.11 Statistical Analysis

All statistical analyses were performed using GraphPad Prism 9. Data are presented as mean ± SEM. For comparisons among more than two groups, one‐way ANOVA followed by Tukey's multiple comparisons test was performed. For pairwise comparisons, an unpaired two‐tailed Student's t test was used. Statistical significance is indicated as P < 0.05; *P < 0.01; **P < 0.001; ***P < 0.0001. For multiple comparisons, groups not sharing a common lowercase letter are significantly different (P < 0.05).

## Author Contributions

W.H. conceived and supervised the project, designed and performed experiments, analyzed the data, and wrote the paper. M.L. (Mao Lin) performed plasmid construction, cell culture assays, low‐oxygen chamber experiments, and animal behavior testing. B.L. performed animal behavior experiments, sample collection in the Qinghai‐Tibet Plateau, and analyzed the data. H.K. and X.X performed the MN substrate fabrication and SEM analysis. H.Y. and Y.W. performed experiments on the Qinghai‐Tibet Plateau and gave advice. Y.Q. performed bioinformatics analysis. M.L. (**Min Li**) performed intraosseous injection and tail vein injection. H.D. performed peripheral blood cell and bone marrow cell isolation and sample collection for RNA sequencing. and flow cytometry analysis. Z.T., X.W., and J.H. performed the chitosan nanoparticle delivery experiment and gave advice. S.C. supervised and performed sample collection of high‐altitude and low‐altitude animals and gave advice. S.D. analyzed the data. T.W. supervised and performed studies on the Qinghai‐Tibet experiment. Y.W. and B.Z. conceived and supervised the project. Q.B. conceived and supervised the project, designed all the experiments, analyzed the data, and wrote the paper. All authors discussed the results and commented on the manuscript.

## Funding

Youth Elite Project of Anhui Academy of Agricultural Sciences (QNYC‐202511); Anhui Province Key research and Development Project (202104e11020003); Natural Science Foundation of Anhui Province (2208085MC51, 2208085MC74); Scientific Research Foundation from the Education Department of Anhui Province, China (KJ2021A0055).

## Conflicts of Interest

The authors declare no conflicts of interest.

## Supporting information




**Supporting File**: advs78002‐sup‐0001‐SuppMat.docx.

## Data Availability

The data that support the findings of this study are available from the corresponding author upon reasonable request.
