# Supplementary material for: MN•CS•IO System: Cross‐Species Study for HBB Function in High‐Altitude Hypoxia Adaptation
Source: Adv Sci (Weinh). 2026 Sep 28:e78002. Online ahead of print. doi: 10.1002/advs.78002 (PMC13618169; doi:10.1002/advs.78002)
Supplement: Supplementary file 1 [file ADVS-9999-e78002-s001.docx]

Supporting Information

MN•CS•IO System: Cross-Species Study for HBB Function in High-Altitude Hypoxia Adaptation

Wenqiao Hui¹²†, Lin Mao²†, Binggang Li²†, Hanyue Kang³†, Zhengquan Tang², Sheng Chen¹, Hui Yang⁴, Yu Qiao¹, Huiqian Ding², Yuhuan Wang⁴, Min Li², Junjie Hu², Shoudong Ye², Xin Wang², Xiaobin Xu³⁵, Yupeng Wang*⁶, Tana Wuren*⁴, Qian Ban*², Baowei Zhang*²

† These authors contributed equally to this work

**Materials and Methods**

***Plasmid construction***

HBB/HBA cDNA and chemical synthesis of DNA were used as templates. Afterwards, primers, with the protective bases added, were designed. The XHOI and XBAI sites of AAV vector were selected for enzyme digestion. After recovering and purifying the product, T4 ligase was used to ligate and amplify the products. The ligated vector was transferred into E. coli, and then coated onto solid medium with ampicillin resistance contained, after culturing overnight, the monoclonal antibodies colony PCR detection was performed to determine whether, they were positive. After that, the positive monoclonal antibodies were isolated and sent to General BioCo., Ltd (Anhui, China) for sequencing. After comparing the sequencing results, the plasmid was constructed.

HBA-A1 and HBB-Flag overexpression plasmid: The codon-optimized cDNA sequences encoding mouse hemoglobin subunit alpha 1 (HBA-A1 protein, encoded by Hba-a1 gene, NCBI accession: NM_008218.2) and plateau pika (*Ochotona curzoniae*) hemoglobin subunit beta (HBB protein, encoded by HBB gene, NCBI accession: XM_040979489.1) were chemically synthesized by General BioCo., Ltd (Anhui, China). For overexpression plasmid construction, the synthesized target gene fragments were separately subcloned into the multiple cloning site (MCS) of the lentiviral expression vector pCDH-CMV-MCS-EF1-copGFP using a restriction enzyme digestion and ligation strategy.

For the plateau pika HBB plasmid construct, a nucleotide sequence encoding a 3×Flag tag (amino acid sequence: DYKDHDGDYKDHDIDYKDDDDK) was fused to the C-terminus of the HBB coding sequence. A 15 bp flexible linker sequence encoding the G4S peptide (amino acid sequence: GGGGS) was inserted between the HBB coding region and the 3×Flag tag. This flexible linker was designed to ensure the correct spatial conformation of each functional domain of the fusion protein and preserve their independent biological activities. In contrast, the mouse Hba-a1 gene overexpression plasmid construct was generated without any epitope tag fusion.

Rragd shRNA: the codon-optimized cDNA sequence of mouse Ras-related GTP-binding protein D (Rragd, NCBI accession: NM_001369055.1) was chemically synthesized by General BioCo., Ltd (Anhui, China). The full-length Rragd cDNA fragment was inserted into the pCDH-CMV-MCS-EF1-copGFP lentiviral vector via the same restriction enzyme digestion and ligation method to construct the Rragd overexpression plasmid.

For Rragd gene knockdown, three independent short hairpin RNA (shRNA) oligonucleotide pairs targeting mouse Rragd were designed and synthesized. The complementary oligonucleotide strands of each shRNA were annealed to form double-stranded DNA fragments. Subsequently, the annealed double-stranded oligonucleotides were ligated into the pLKO.1-EGFP-puro lentiviral RNA interference (RNAi) vector, which was pre-digested with *Age*I and *Eco*RI restriction enzymes, to generate Rragd shRNA recombinant plasmids.

***Co-immunoprecipitation assay for detecting interaction between exogenous HBB and mouse HBA-A1***

HEK293T ***Cell culture and transfection***

HEK293T cells (ATCC) were cultured in high-glucose DMEM medium (Gibco) supplemented with 10% fetal bovine serum (Gibco) and 1% penicillin-streptomycin solution. Cells were routinely passaged and maintained in a humidified incubator at 37 °C with 5% CO₂. Prior to the co-immunoprecipitation assay, HEK293T cells were seeded in 10 cm culture dishes and cultured until reaching 70%-80% confluence. Cells were co-transfected with *Hba-a1* and HBB-Flag plasmids at a 1:1 mass ratio using M5 HiPer Lipo2000 Transfection Reagent. Meanwhile, cells transfected with an equal amount of empty vector were set as the negative control group. After transfection, cells were continuously cultured for 48 h before collection for subsequent protein interaction detection.

***Total protein extraction and whole-cell lysate preparation***

After transfection, the culture medium of HEK293T cells was discarded, and the cells were washed twice with pre-cooled phosphate-buffered saline (PBS). For each culture dish, 1 mL of pre-cooled non-denaturing lysis buffer (50 mM Tris-HCl, pH 7.4, 150 mM NaCl, 1 mM EDTA, 1% NP-40) supplemented with a protease inhibitor cocktail (Proteintech, Cat No. PR20035) was added. The cells were lysed by gentle shaking at 4 °C for 30 min. Subsequently, the cell lysates were centrifuged at 12,000 × g for 15 min at 4 °C to remove cell debris, and the supernatants were collected as total protein lysates. In total, 100 µL of the total lysate was reserved as the input sample, mixed with 20 µL of 5× SDS loading buffer, and denatured by boiling at 100 °C for 10 min for subsequent use. Additionally, 40 µL of the total lysate was taken to quantify the total protein concentration via the BCA method. The remaining approximately 900 µL of lysate was retained for the subsequent co-immunoprecipitation (Co-IP) assay.

***Co-immunoprecipitation (Co-IP) assay***

To reduce non-specific binding background, the remaining cell lysate was pre-cleared by incubation with 30 µL of Protein G agarose purification resin (YEASEN) with gentle rotation at 4 °C for 1 h. After incubation, the mixture was centrifuged at 8,000 × g for 3 min at 4 °C, and the supernatant was carefully transferred to a new centrifuge tube while the agarose bead pellet was discarded.

The pre-cleared supernatant was incubated with 30 µL of Anti-DYKDDDDK (Flag) Affinity Gel (YEASEN) with slow rotation at 4 °C for 2 h to specifically capture HBB-Flag protein and its interacting protein complexes. After incubation, the affinity gel beads were harvested by centrifugation and washed three times with TBS buffer to thoroughly remove unbound contaminating proteins. After the final wash, the supernatant was completely aspirated. The gel beads were resuspended in 150 µL of 1× SDS loading buffer and boiled at 100 °C for 10 min for protein denaturation. The eluate was collected by centrifugation as the Co-IP sample for subsequent Western blot analysis.

***Western Blot Analysis***

Equal amounts of protein from input and Co-IP samples were separated by 15% sodium dodecyl sulfate-polyacrylamide gel electrophoresis (SDS-PAGE) and wet-transferred onto polyvinylidene fluoride (PVDF) membranes (Millipore). The membranes were blocked with 5% (w/v) skimmed milk in TBST at room temperature for 1 h and then incubated with primary antibodies overnight at 4 °C. The primary antibodies used were as follows: anti-Flag tag rabbit monoclonal antibody (ABclonal, AE169PM) and anti-mouse Hba-a1 rabbit polyclonal antibody (ABclonal, A9293). After thorough washing with TBST, the membranes were incubated with horseradish peroxidase (HRP)-conjugated secondary antibody (goat anti-rabbit IgG, Proteintech, RGAR301) at room temperature for 1 h. Finally, protein bands were visualized using an ultra-sensitive ECL chemiluminescent substrate (Proteintech), and images were captured using the Tanon imaging system (Bio-Rad).

***K562 cell culture***

K562 cells were purchased from Cell Bank, Shanghai Institute of Biochemistry and Cell Biology, Chinese Academy of Sciences, Shanghai, China. K562 cells were cultured in a humidified atmosphere of 5% CO_2_ in RPMI1640 (Gibco), supplemented with 10% fetal bovine serum (FBS), and 1% penicillin/streptomycin mixture (P1400, Solarbio, China). Cells were then seeded on a 6-well cell plate with a density of 2×10^6^. The plates were incubated at 37℃ in a humidified 5% CO_2_ incubator for 3days, during which, K562 cells received RPMI1640 (Gibco) condition medium, and underwent four passages before the following experiments.

For cells seeding on MN substrate in commercial plates, 2×10^6^ cells were seeded on the MN substrate placed in six-well plates. They were then cultured with above method. In the subsequent experiments, K562 cells were subjected to different treatments, including electroporation, Chitosan.

***Electroporation***

2×10^6^ K562 cells were harvested and centrifugated at 800rpm for 4 min. Then, K562 cells were resuspended in 100 μL of the desired buffer (SCOTOP SCIENTIFIC Electroporation Buffer), followed by the addition of 4 μg plasmid. After that, the mixture was transferred to a sterile 0.2-cm cuvette and electroporated via a Lonza® Nucleofector® II electroporation system (900V, 30 ms, 1 pulse). After electroporation, the transfected K562 were harvested, and gently resuspended in 2 mL of pre-warmed medium and were then seeded in cell plates and cultured at 37 ℃ and 5% CO_2_. Subsequently, at 12h, 24h, 48h, and 72h post-transfection, cells were observed under an inverted microscope (Leica DMI8) at each corresponding time point.

***Preparation of Chitosan***

Chitosan (CS) was purchased from Shanghai Sangon Biotech, China (Cat. # A600614-0500). Four mg chitosan was dissolved in 20 mL of 0.2 mg/mL acetic acid solution. The HBB or HBA plasmid was dissolved in a 5 mM sodium sulfate solution. Both solutions were adjusted to a pH of 5.5 with 10 M NaOH, and preheated in a water bath at 55 °C for 15 minutes. Afterwards, the two solutions were mixed in equal volumes and vigorously stirred for 30s, followed by a 30-minute incubation, to form nanoparticles with an N/P ratio ranging from 1 to 20.

***Chitosan characterization***

The diameters (particle size distribution) and zeta potentials of CS were analyzed by using the Zetasizer Nano ZS (Malvern, Instruments UK) in conjunction with dynamic light scattering (DLS), with the concentration of SC maintained at 0.1 mg mL-1 in ddH_2_O. And each sample was replicated over 3 runs. Samples with the concentration of 0.1mg/mL were cast onto a silicon chip. 20 μL of the resuspended samples were added dropwise to 200-mesh grids and incubated at room temperature for 10 min, then the grids were negatively stained with 2% phosphotungstic acid for 3 min, and the remaining liquid was removed by filter paper, and then observed with a JEM1400 transmission electron microscope.

***Chitosan delivery into cells***

2×10^6^ cells were seeded on the six-well plates, K562 cells were firstly starved in serum-free RPMI medium in six-well plates for 6 hours, and then centrifuged to remove medium at the rate of 800×g for 5 min. After that, 4 μg plasmid was added and cells were co-incubated with plasmid for 6h. Afterwards, cells were collected and centrifuged at 800×g for 5min. The cell pellet was then transferred to a new six-well plate. 2 mL of RPMI1640 supplemented with 10% FBS was then added to each well.

***MN•CS delivery into cells***

2×10^6^ cells were seeded on the MN substrate placed in six-well plates, and subjected for Chitosan delivery into cells (the above method).

***Cell viability detection***

Electroporation cells, CS delivery cells and cells without any treatment were harvested and evaluated using the CCK-8 assay according to the manufacturer’s instructions. K562 Cells were seeded into 96-well plates, with 100 μL RPMI1640 supplemented with 10% FBS added for each well, to adjust the cell density to 5×10^3^ each well. Then 10 μL CCK-8 solution (APExBIO) was added to each well and incubated at 37°C. At 0 h, 12 h, 24 h, 36 h, and 48 h post incubation, optical density was measured (at 450 nm) through a microplate reader (SpectraMaxi3 Platform, Molecular Devices, Austria).

***qPCR***

RNA was extracted from the cells with TRIzol (Life Sciences, USA). One μg RNA was reversely transcribed for cDNA, using the SuperScript III first-strand synthesis kit. The qPCR analysis was performed using PowerUp SYBR Green Master Mix (Applied Biosystems, California) and primers as described Table 4.

***Plasmid copy number detection***

A series of diluted samples were prepared from plasmids of known concentrations to yield target plasmids with varying concentrations. A standard curve was plotted, correlating the Ct values (fluorescence signal threshold period) of standard plasmid samples with their respective known concentrations.

DNA was extracted from cells after 48h electroporation using TRIzol (Life Sciences, USA). A mixture was prepared consisting of 1 µL of Phanta Max ultra fidelity DNA polymerase, 25 µL of 2 × Phanta Max buffer, 2 µL of 10 µM primers, 1 µL of 10 mM dNTP mixture, and 2 µL of genomic DNA. To complete the PCR reaction, ddH2O was added to make the total volume to 50 µL, and the sequence was then aligned with the correct sequence.

qPCR analysis was performed utilizing PowerUp SYBR Green Master Mix (Applied Biosystems, California) along with primers (Table 4). The data was calculated through standard curves, and then the copy number of cell plasmids was obtained.

***Flow cytometry detection for plasmid transfected K562 cells***

K562 cells were centrifuged with 500×g for 5 minutes. After carefully removing the supernatant, cells were resuspended in 500 μL PBS, and centrifuged, again. Cells were then resuspended with 500 uL PBS to adjust the concentration to 1×10^6^ cells/mL. Cell suspension was then transferred to a flow cytometry (BD-FACSCalibur) and performed analysis, using FL2 channel to detect tdTomato.

***Attach Lin^+^ antibody onto silica surface.***

First, aminosilylate the substrate Surface. Following thoroughly washed and dried thesubstrate, 2% solution of the Aminosilane Reagent (3-Aminopropyltriethoxysilane) were prepared. The substrate surface was then immersed in the reagent for 30 seconds. After that, the surface was rinsed with anhydrous acetone and air-dry was allowed. Second, maleimide-activate the amino-modified surface. 2 mg Crosslinker (Sulfo-SMCC) was added to 1 mL coupling buffer. The silylated surface was covered with the crosslinker solution, and then incubated for 1 hour at room temperature. Then, the modified surface was rinsed by coupling buffer. Third, sulfhydryl addition with Traut’s reagent. Dissolve 4 μg antibody Lin^+^ in 475 µL of coupling Buffer. 2 mg Traut’s Reagent was dissolved in 1 mL of the pH-adjusted Coupling Buffer. 25 µL Traut’s Reagent solution was immediately added to the antibody solution, followed by a 45-minute incubation at room temperature. The fourth step is cross-link sulfhydryl-containing antibody to activated surface. Cover the maleimide-activated surface material with the antibody solution. After incubation for 2-4 hours at room temperature, the reaction solution was removed. After that, the surface was thoroughly rinsed by coupling buffer to ensure that only covalently attached antibody molecules remain.

***Donor Lin***^-^ ***cells isolation***

First, a cocktail of anti-lineage antibodies was attached onto MN silica surface (Methods were shown in Supplementary methods). The antibodies include anti-CD3 (17A2), anti-CD45R (B220), anti-CD11b (M1/70), anti-TER119 (TER, and anti-Ly-G6 (Gr-1) (mouse hematopoietic lineage antibody, clone17A2# 22-7770-72, 1:500, Thermo Fisher). Then, MN substrates with Lin antibody attachment were washed with PBS twice for each. Afterwards, they were placed in the six-well plates for donor bone marrow cells seeding.

Donor bone marrow cells isolation: Donor mouse was euthanized with 2% isoflurane inhalation, via the anaesthesia equipment (ZS-MV-IV, Beijing Zhongshi Dichuang Technology Development Co., Ltd., China), with an oxygen flow rate of 0.41mL/min (at a fresh gas flow of 4L/min) for 2 minutes followed by cervical dislocation. After disinfecting the animal with 70% ethanol, an incision was made along the hindlimb to expose the tibia. Then, the surrounding soft tissues were carefully removed to fully expose the bone. After carefully cutting through the connective tissues at both ends of the tibia to free the bone, the connective tissues were completely removed from the tibia. The fresh isolated tibia was then placed on ice and rinsed in pre-cooled PBS supplemented with 2% FBS to isolate the bone marrow. Bone marrow tissues were dispersed by repeatedly blowing and suctioning until there were no observable tissue blocks. Samples were centrifuged at 500×g for five minutes at 4°C. After discarding the supernatant, cells were resuspended in 1 mL red blood cell lysis buffer (Beyotime c3702). Two minutes later, 1 mL PBS supplemented with 2% FBS were added to stop cell lysis. Cells were then centrifuged at 500×g for five minutes. Finally, the supernatant was discarded to harvest bone marrow cell pellet.

10^6^-10^7^ bone marrow cells from donor mice were directly seeded on MN substrates (with Lin^+^ antibody attachment) placed in the cell plates and cultured in 600 μL RPMI-1640 supplemented with 10% fetal bovine serum (FBS) and 100 U/mL penicillin-streptomycin in each well. Cells were subsequently cultured in a humidified atmosphere of 5% CO_2_ incubator at 37℃. Ten minutes later, the supernatant was carefully aspirated using a 1 mL pipette, and the suspension Lin^-^ cells were harvested. The harvested Lin^-^ cells were used for subsequent foreign plasmid transfection.

***SEM characterization of Lin^+^ Cells attached onto MN substrate***

Lin^+^ cells attached onto the MN substrate were allowed to rest for 20 min in a light-shielded environment. Subsequently, surface of the substrate was meticulously cleansed with PBS solution, followed by immersion in a 4% paraformaldehyde solution for 30 min to ensure proper fixation. Then, the MN substrate underwent a gradual dehydration process by sequential immersion in ethanol solutions of increasing concentrations (30%, 50%, 70%, 85%, 95%, and finally 100%), each step lasting 5 min. Once thoroughly dehydrated, the chip was placed in a fume hood for drying. Finally, a thin layer of gold was sputtered onto its surface, preparing it for observation with SEM (JEOL JSM-6060LV).

***Preparation of Animal HBB and HBA plasmid for Lin***^-^ ***cells transfection***

Samples for Qinghai-Tibet Plateau mammals: Blood samples collected from Tibetan sheep and Tibetan pig were from farm located in Gonghe County (at an altitude of 3000 m), Hainan Tibetan Autonomous Prefecture, Qinghai Province, China. The collected blood samples were immediately stored in dry ice and transported to the lab, for DNA isolation and PCR amplification for targeted HBB and HBA gene. HBB and HBA gene for plateau pika, Wild Yak and Root vole, with genomic sequence available, were chemically synthesized by General Bio Co., Ltd (Anhui, China).

Samples for lowland mammals: Blood samples of Hu sheep were collected from a farm located in Changfeng county (at an altitude of 30 m), Hefei, Anhui, China. HBB and HBA gene for other lowland species, including cattle, pig, rabbit, and house mouse, with genomic sequence available, were chemically synthesized by General Bio Co., Ltd (Anhui, China).

***PCR amplification and validation of chemical synthesis of HBB and HBA***

The primers were designed and synthesized by Sangon Bicotech Co, Ltd (Shanghai, China). The detailed Primer sequences were listed in Table 3. PCR amplification was used as follows: 1 µL of DNA Polymerase, 25 µL of 2×Phanta Max buffer, 2 µL of 10 µM corresponding primers set, 1 µL of 10mM dNTP, 2 µL of DNA, and 19 µL ddH2O added to make up the 50 µL full reaction volume. Samples were run on the GeneAmp® PCR System 9700 thermal cycler. Gene expression of HBB and HBA were detected by performing qPCR at 95℃ for 3 min, followed by 35 cycles of 95℃ for 15 s, 64-66℃ for 15 s, and 72℃ for 30s), 72℃ for 5 min. Resulting PCR amplicons were analyzed by electrophoresis in 1.5% agrose gel. PCR products were then performed for Sanger Sequencing. The sequences were analyzed by DNAMAN version 8.0 (LynnonBiosoft, Quebec, Canada), and the nucleotide was blasted by the National Center for Biotechnology Information (NCBI).

***CS delivery into Lin^-^cells***

The respective target HBB and HBA plasmids were delivered into Lin^-^ cells through CS. Bone marrow cells of donor mouse were seeded in six-well plates placed with MN substrate at a density of 2×10^6^ cells/well. After 15 minutes, the supernatant was carefully aspirated using a 1 mL pipette, the harvested Lin^-^ cells suspension was collected into new six-well plates and starved with 2 mL of serum-free media for 6h (culture conditions of 37℃ and 5% CO_2_). Then, the supernatant was added to 2 mL of fresh RPMI-1640 and FBS as well as 10 µg CS together with 4 µg HBB plasmid mixed solution, which was then incubated in the incubator for 6 hours. The control group was set with only 4 µg of HBB plasmid added and cells were treated in the same manner. In the subsequent HBB plasmid from animals experiments transfection, the CS group was divided into four subgroups: five high-altitude animal HBB plasmids, five low-altitude animal HBB plasmids, five high-altitude animal HBA plasmids, and five low-altitude animal HBA plasmids, with a total of 20 different constructs (3 biology replicates, 60 wells). Cells cultured on MN but without any transfection operation served as the control group.

***Flow cytometry detection***

Cell suspension was collected to the centrifuge tube, and centrifuged at 500×g for 5 minutes. After removing the supernatant, the cell pellet was resuspended with 1 mL of PBS to adjust the concentration to 1×10^6^ cells/mL. After repeating the centrifugation, cells were resuspended in 500 µL of serum containing PBS at 1×10^6^ cells, and then transferred to a flow cytometry (BD-FACSCalibur) to detect the tdTomato signal using the FL2 channel. Cells were gated on physical parameters in FSC vs SSC. Cell debris and dead cells with the lower FSC signals and located in the lower left corner of the FSC vs. SSC scatter plot (less than 400) were excluded. Data was analyzed using FlowJo V.10.

All the groups were subjected to the following detection: fluorescence microscopy, Real-time PCR, and plasmid copy number detection, were consistent with those used in the K562 cell experiments. Methods were shown in Supplementary methods.

***Behavior testing***

*Open-Field Test*

Exploratory activity and anxiety-like behavior were measured using an open-field apparatus (50×50×40cm), with camera hanged above the apparatus to ensure the field of view completely covering the mine setting. Before the experiment, all mice were placed in the experiment room for 10 minutes to acclimate to the environment. When began the experiment, mice were placed in the central area of the apparatus, with its back facing away from the experimenter, and behavior was record for 5 minutes. At the end of 5 minutes test, mouse was returned to its home cage. The experimental apparatus was cleaned to remove any residue from previously tested mouse, such as urine, feces, and odor. After air dry, repeating the test procedure for the new mouse. The recorded data were analyzed using Visu Track software, with behavioral indicators such as locomotor activity and central stay time assessed.

*Treadmill test*

Before the formal experiment, mouse was performed for 4 days of adaptive treadmill training, three times for one day, with an interval of 10 minutes between each training session. The treadmill speed was set at 12m/min. For the formal experiment, after activating the treadmill and video recording devices, mouse was placed gently and carefully on the treadmill to reduce stress, followed by adjusting the parameters of treadmill settings at 30 minutes for duration and 12m/min for the speed, then start button was clicked. Upon finishing the 30 minutes training, mouse was carefully removed from the treadmill, and replaced with the next one. Data including behavior, the times of falling off the treadmill, was recorded and analyzed.

*Y-Maze Test*

Spatial working memory and exploratory activity were measured by Y-maze experiment. The materials include a Y-maze apparatus (arm length: 30 cm, arm bottom width: 6 cm, arm upper width: 15 cm), partitions to block access to one arm during the initial phase, and the video recording equipment for capture behavior. Before the experiment, both Y-maze and experiment mice were cleaned with alcohol swab. Then, mouse was placed in the center arms of the Y-maze, with its back facing to the open arms, and allowed to explore freely for 10 min. Then, return the test mouse into its home cage. The partition for blocking access to the new arm was removed, and access was allowed to all three arms. One hour later, the test mouse was repositioned to the center arm of the Y-maze, at the same starting place in the initial phase. The mouse behavior was recorded using the video equipment for 5 minutes, allowing the free exploration into all three arms of the maze. At the end of the exploration of the maze, the test mouse was removed and returned to its home cage, while the maze was cleaned with alcohol for the next trail preparation. VisuTrack software was used to analyze the record videos. The mouse movement was tracked and the numbers of entries into each arm, as well as time spent in each arm were calculated.

*Novel Object Recognition (NOR) Experiment*

NOR experiment was used to evaluate cognitive memory capabilities in mice. The material includes an arena (50×50×40cm), three identical objects (A, B, and C). Video recording equipment was placed to track mouse behavior. The NOR experiment involves in three phases: First, for the adaptation period: mice were placed to the empty box and allowed to move freely for 10 minutes to adapt to the experiment environment. Second, for the familiarization period: Two objects, A and B, with a distance of 10 cm from the side walls, were placed in the apparatus, Mouse were then placed in it, with its back facing the object at an equal distance, and allowed for explore freely for 5 minutes. Mouse contacted the objects with its mouth or nose, or approaching the objects within about 2-3 cm is considered as exploration. Within five minutes, the number of times, duration, and distance that mouse explored the objects were assessed. One hour later, after replacing object A with C in the apparatus, the mouse was placed in the open-field, with its back facing the object at an equal distance, and allowed for explore freely for 5 minutes. Similar to the last phase, video was captured and the number of times that mouse explored the objects were assessed.

*Tail-suspension test*

Each mouse was suspended by the tail 30 cm above the floor of the chamber (31×15×30cm) with adhesive tape placed 1 cm from the tip of the tail. The video camera was placed at a level that aligned with the mouse body height, to ensure the entire view field was captured in the video frame. After recording the mouse behavior for 6 minutes, especially focusing on the last 4 minutes immobility exhibited by mouse, the video was analyzed.

**Supplementary Text**

**Figures S1 to S10**

***Figure. S1 Process of MN substrate fabrication and detection of K562 cells transfected with foreign plasmids and comparison with the conventional electroporation method.*** *A, Process of MN substrate fabrication. B, tdTomato red fluorescence microscopy detection of K562 cells transfected with human HBB plasmid via four different methods: MN, CS, MN•CS and Electroporation. Scale bars, 20 μm. Results shown as mean ± SEM, n=3. C, Flow cytometry detection of K562 cells transfected with human HBB plasmid via four different transfection methods: MN, CS, MN•CS and Electroporation. Results shown as mean ± SEM, n=3. D**, E. Relative expression of foreign human HBB (D), and the average number of HBB plasmids within single cell (E) in K562 cells with different transfection methods: MN•CS system and electroporation. Results shown as mean ± SEM, n=3. An independent samples t-test was employed for the group comparison. P>0.05. ns: not significant. F, The proliferation curve depicting the viability of K562 cells after MN·CS system treatment or electroporation.* *Results shown as mean ± SEM, n=3.*

***Figure. S2 Comparative analysis of HBB and HBA protein structure predication and amino acid sequence alignment in different animals.*** *A, HBB amino acid sequence alignment in different animals. B. Comparative analysis of HBB protein structure predication across different animals. C, HBA amino acid sequence alignment in different animals. D. Comparative analysis of HBA protein structure predication across different animals. E. Interaction between HBB-Flag and Hba-a1.293T cells co-transfected with HBB-Flg, Hba-a1 or empty vector were subjected to anti-FLAG-based co-immunoprecipitation (Top panel). Input and IP fractions were immunodetected by FLAG, Hba‑a1 and β‑Tubulin antibodies. Β-Tubulin acts as input loading control.*

***Figure. S3 Flow cytometry detection of red fluorescence Lin^−^ cells after transfection with HBB and HBA plasmid from different animals, using the FL2 channel.*** *Low-altitude animals (Left Panel), High-altitude animals (Right Panel).*

***Figure. S4 Microscopy detection of tdTomato red fluorescence in Lin^−^ cells after transfection with HBB and HBA plasmid from different animals.*** *A. Cell counting for tdTomato^+^ Lin^−^ cell population (red fluorescent signal), also means Lin^−^ cells successfully transfected with HBB and HBB+HBA from different animal species, as determined by fluorescence microscope. B. Microscopy detection of tdTomato red fluorescence, with the average efficiencies of 91.90% for HBB and 92.51% for HBA. Scale bars for transfection group, 20 μm. Scale bars for Control, 30 μm.*

***Figure. S5 Intracellular duration of plasmid. A-E.*** *Time-dynamic detection of human HBB in Lin^−^ cells of recipient mice following transfection with human HBB plasmid. Fluorescence microscopy images (A) and quantitative analysis (cell counting) (B) of tdTomato fluorescence, dynamic tdTomato expression (C) and HBB expression (D) determined by qPCR, and the average number of HBB plasmids within a single cell (E). Results shown as mean ± SEM. n=3. g, Scale bars, 25 μm.*

***Figure. S6 Investigation into the typical range of Lin^-^ cells injected per recipient mouse and comparison of the two methods (intraosseous and tail vein injection methods) for transplantation mouse model establishment.*** *A, B, Investigation into the typical range of Lin^−^ cells injected per recipient mouse. Fluorescence microscopy detection of the bone marrow cells from the different number of donor mice. Scale bars, 30 μm (A). Relative expression of human HBB in the bone marrow cells and peripheral blood cells of recipient mice at 1d and 7d post injection of Lin^−^ cells originated from the different number of donor mice, and transfected with human HBB plasmid (B). C, D, Comparison of the two methods: intraosseous injection (Left Panel) and tail vein injection (Right Panel) for transplantation mouse model establishment. D. Fluorescence microscopy detection of bone marrow cells isolated from recipient mice, which were injected with Lin^−^ cells following transfection with the human HBB plasmid, via intraosseous and tail vein injection methods. Scale bars, 40 μm. E, Relative expression of human HBB in bone marrow cells and peripheral blood, isolated from recipient mice that were injected with Lin^−^ cells following transfection with the human HBB plasmid, via intraosseous and tail vein injection methods.* *n=3 per group. Results shown as mean ± SEM. F, G, Dynamic detection of transfected human HBB in recipient mice transplantation of Lin^−^ cells with human HBB transfection during the injection period of days 7-30. Relative expression of human HBB (F), and average number of human HBB plasmids within a single cell (G). H. Immunology factors detection in recipient mice after transplantation of Lin^−^ cells with HBB from high/low altitude animals. n=3 per group. Results shown as mean ± SEM. ns: not significant*

***Figure. S7 cross-species HBB transplantation mice in low oxygen chamber followed by behavioral test A.*** *Workflow model of cross-species HBB transplantation mice experiment in the low oxygen chamber. B. The number of falls in the treadmill experiments of the groups. C. Behavioral testing in xenogeneic HBB-transplanted mouse models in the open-field test. C1. Representative heatmaps and Trajectory maps mice in the open-field test. C2. Movement speed detection. C3. Time in center zone. C4. Immobility time. B-C. Results shown as mean ± SEM, n=8, P values were calculated using one-way ANOVA. In the bar chart, different letters indicate significant differences between the groups.*

***Figure. S8 Transcriptome analysis of the peripheral blood cells and bone marrow cells with foreign HBB recipient mice (transplantation of Lin- cells transplanted HBB from high-altitude and low-altitude animals) in low oxygen chamber, and un-transfected mice under both hypoxic (LOC) and normoxic conditions (Control).*** *A-B. GO (A) and KEGG (B) analysis of differential expression genes in Peripheral blood cells of different groups. C-D. GO (C) and KEGG (D) analysis of differential expression genes in bone marrow cells of different groups.*

***Figure. S9 Gene expression analysis of Pika-HBB transplantation mice in hypoxic conditions using volcano plots, clustering, and functional enrichment.*** *A, B. Volcano plots analysis of differential expression genes in both peripheral blood cells (A) and bone marrow cells (B) of the three groups: C-D. Cluster (C), Go (D) and KEGG (E) analysis of differential expression genes in the peripheral blood cells of the two groups: Pika-HBB transplantation mice in the hypoxic conditions and normoxic conditions. F-H. Cluster (F), Go (G) and KEGG (H) analysis of differential expression genes in the bone marrow cells of the two groups: Pika-HBB transplantation mice in the hypoxic conditions and normoxic conditions.*

***Figure S10. Validation of RNAi and overexpression plasmids designed for Rragd.*** *A, Detection of shRNA knockdown efficiency.* *Among the three designed Rragd shRNA plasmid, shRNA-3 exhibited the highest knockdown efficiency. Therefore, shRNA-3 was selected for the subsequent animal experiments. B, Detection of Rragd overexpression efficiency. Experiments were performed in C2C12 cells.* *The overexpression plasmid significantly upregulated the mRNA expression level of* Rragd.

***Figure. S11. Behavioral assessments of transplantation mice following Rragd overexpression and knockdown.*** *A, Open‑field test. Quantification of time spent in the center zone (Left), average speed (Middle), and immobility time (Right). B, Y-maze test.* *Spontaneous alternation percentage. C, Number of falls in the tread-mill test. D, Immobility time in the tail suspension test.*

***Figure. S12 Histology analysis of brain tissue of cross-species xenogeneic HBB-transplanted mouse models on the Qinghai-Tibet plateau (QTP).*** *A, Hematoxylin and Eosin (HE) analysis of the brain tissue within recipient mice injection of Lin^-^ cells, following transfection with HBB plasmids from ten different animals. Scale bars, 2000 μm. B, Immunofluorescence of the brain tissue of within recipient mice injection of Lin^-^ cells, following transfection with HBB plasmids from ten different animals and transported to the Qinghai-Tibet plateau. Scale bars, 2000μm. Groups in A and B: Control (Plateau): Mice without HBB transfection, which were transported to and housed on the Qinghai-Tibet plateau with the altitude of 3,800 meters. Control (Plain): Mice without HBB transfection and housed in normoxic conditions of Plain. sheep-L: recipient mice transplanted with HBB of low-altitude sheep, sheep-H: recipient mice transplanted with HBB of high-altitude sheep. The same applies to other animals. All transplantation mice were transported to and housed on the Qinghai-Tibet plateau with the altitude of 3,800 meters.*

***Figure. S13 Bone marrow transplantation mice model establishment and sequencing results of HBB gene editing at ROSA 26 site.*** *A. Flow cytometry analysis of CD45.1+ transplanted cell proportion in recipient mice at 6 weeks post transplantation, and comparison with untreated controls. B. Treadmill test to assess the number of mice fell off the treadmill during the test in the bone marrow transplantation mice and comparison with controls (n=8). C. Schematic diagram of Pika HBB gene insertion into ROSA26 locus in bone marrow transplantation mice. D. Electrophoretic detection image of two DNA fragments, specifying the integration of the HBB/GFP into the ROSA 26 site, in the bone marrow transplantation mice at 12-wks post transplantation. E. Amplicons sequence in (D) to confirm the correct integration of the HBB/GFP at the boundaries of 5’- and 3’-junctions, in the bone marrow transplantation mice at 12-wks post transplantation.*

**Data Tables**

**Table S1 HBB and HBA sequence information for ten animals and human being**

| Species (breed) | Abbreviation | NCBI ID (HBB) | NCBI ID (HBA) |
| --- | --- | --- | --- |
| Ovis Aries (Hu Sheep) | Sheep-L | NM_001097648 | XM_015103978 |
| Ovis Aries (Tibetan Sheep) | Sheep-H | GCA_017524585.1 | GCA_017524585.1 |
| Sus Scrofa (Domestic pig) | Pig-L | GCA_029890215.1 | GCA_029890215.1 |
| Sus Scrofa (Tibetan Pig) | Pig-H | GCA_000472085.2 | GCA_000472085.2 |
| Bos Taurus (Cattle) | Cattle-L | NM_173917 | NM_001077422 |
| Bos Mutus (Wild Yak) | Yak-H | XM_005907921 | XM_014483170 |
| Oryctolagus Cuniculus | Rabbit-L | NM_001082260 | NM_001082389 |
| Ochotona Curzoniae | Pika-H | XM_040979489 | XM_040973671 |
| Mus Musculus (House mouse) | Mouse-L | NM_001278161 | NM_008218 |
| Microtus Oeconomus | Vole-H | GCA_963919705.1 | GCA_963919705.1 |
| Homo Sapiens | Human | NM_000518 | / |

**Table S2 Blood indices of recipient mice that were injected with Lin^-^ cells after transfection with exogenous HBB or HBB+HBA plasmid, derived from animals in high-altitude Tibetan species and their low-altitude counterparts**

|  | WBC | Neu% | Lym% | Mon% | Bas% | PLT | MPV | MFR | HFR | RHE |
| --- | --- | --- | --- | --- | --- | --- | --- | --- | --- | --- |
| Sheep-L-B | 5.17±0.50 | 33.70±0.86 | 65.7±3.64 | 3.05±0.40 | 1.21±0.15 | 381.33±56.66 | 6.96±0.14 | 27.87±1.03 | 4.90±0.33 | 16.10±0.67 |
| Sheep-H-B | 4.90±0.33 | 34.40±0.86 | 59.70±2.49 | 2.18±0.15 | 1.41±0.08 | 289.67±69.86 | 6.89±0.50 | 30.23±1.38 | 5.30±0.94 | 16.27±0.45 |
| Pig-L-B | 5.52±0.19 | 36.73±0.70 | 61.43±4.99 | 2.96±0.27 | 1.37±0.18 | 286.33±57.74 | 7.03±0.47 | 25.40±4.00 | 5.47±0.56 | 15.90±0.41 |
| Pig-H-B | 5.42±0.27 | 31.57±0.58 | 59.50±4.33 | 2.73±0.52 | 1.56±0.12 | 305.67±71.26 | 6.70±0.09 | 25.33±3.07 | 5.27±0.95 | 16.23±0.82 |
| Cattle-L-B | 4.77±0.22 | 31.03±0.83 | 53.10±1.59 | 2.84±0.32 | 1.38±0.21 | 367.67±52.26 | 6.70±0.32 | 24.57±2.27 | 4.70±0.16 | 16.47±0.05 |
| Yak-H-B | 5.03±0.04 | 32.47±0.87 | 62.30±4.99 | 2.90±0.55 | 1.53±0.23 | 320.00±34.22 | 6.44±0.12 | 28.47±4.22 | 4.77±0.34 | 16.77±0.49 |
| Rabbit-L-B | 5.45±0.40 | 32.73±0.87 | 63.93±4.91 | 3.25±0.24 | 1.45±0.17 | 222.67±72.53 | 6.75±0.24 | 28.10±1.77 | 4.47±0.05 | 16.00±0.22 |
| Pika-H-B | 5.18±0.33 | 35.93±0.40 | 60.27±4.99 | 2.70±0.40 | 1.55±0.30 | 289.00±130.72 | 7.11±0.75 | 24.70±4.68 | 5.23±0.90 | 15.27±0.25 |
| Mouse-L-B | 4.88±0.10 | 38.43±0.45 | 66.70±10.22 | 2.45±0.27 | 1.58±0.27 | 337.00±57.98 | 6.85±0.56 | 26.00±3.13 | 5.73±0.57 | 15.77±0.21 |
| Vole-H-B | 5.15±0.30 | 35.87±0.52 | 65.03±5.58 | 2.90±0.64 | 1.28±0.18 | 303.67±52.49 | 6.30±0.10 | 27.73±4.12 | 4.70±0.54 | 15.80±0.79 |
| Sheep-L-AB | 5.31±0.18 | 36.90±0.22 | 70.73±5.24 | 2.91±0.46 | 1.49±0.14 | 359.00±100.44 | 7.01±0.17 | 27.40±2.84 | 5.57±1.03 | 17.10±0.51 |
| Sheep-H-AB | 4.37±0.26 | 41.30±0.45 | 63.23±2.75 | 2.97±0.62 | 1.25±0.05 | 286.00±65.26 | 6.93±0.54 | 27.53±4.07 | 5.67±0.37 | 16.03±0.48 |
| Pig-L-AB | 5.30±0.13 | 39.93±1.11 | 57.37±6.86 | 2.97±0.30 | 1.62±0.06 | 336.00±86.92 | 6.80±0.46 | 29.57±1.86 | 5.90±0.00 | 16.47±0.48 |
| Pig-H-AB | 4.31±0.17 | 35.87±0.74 | 59.73±4.75 | 3.05±0.30 | 1.49±0.26 | 381.33±42.71 | 7.05±0.43 | 28.47±1.68 | 4.93±0.42 | 16.23±0.49 |
| Cattle-L-AB | 4.48±0.33 | 40.33±0.62 | 56.80±5.42 | 2.64±0.44 | 1.50±0.05 | 328.00±101.91 | 6.70±0.26 | 30.93±1.05 | 4.80±0.71 | 15.27±0.17 |
| Yak-H-AB | 4.92±0.19 | 32.17±0.50 | 64.30±10.67 | 2.19±0.24 | 1.64±0.18 | 337.33±123.16 | 6.88±0.37 | 24.63±0.26 | 5.10±0.08 | 16.37±0.46 |
| Rabbit-L-AB | 5.28±0.15 | 36.10±0.54 | 67.27±7.76 | 2.85±0.21 | 1.46±0.09 | 348.33±57.93 | 7.26±0.56 | 27.80±4.55 | 5.40±0.91 | 15.93±0.66 |
| Pika-H-AB | 4.98±0.24 | 36.80±0.90 | 62.50±6.67 | 2.87±0.58 | 1.25±0.09 | 276.33±66.04 | 6.55±0.28 | 26.33±4.66 | 5.57±0.93 | 16.57±0.21 |
| Mouse-L-AB | 5.26±0.10 | 32.03±0.21 | 62.93±4.95 | 2.51±0.62 | 1.46±0.19 | 259.67±63.62 | 6.47±0.38 | 27.87±4.29 | 4.97±0.61 | 16.13±0.77 |
| Vole-H-AB | 4.32±0.19 | 33.57±0.87 | 58.83±5.02 | 2.92±0.45 | 1.32±0.11 | 328.67±32.89 | 6.64±0.52 | 26.90±3.54 | 5.83±0.29 | 16.00±0.64 |
| LOC | 4.53±0.24 | 34.60±0.45 | 60.93±6.87 | 2.27±0.07 | 1.50±0.11 | 372.00±32.87 | 6.89±0.34 | 28.43±2.37 | 2.97±0.60 | 16.53±0.31 |
| Control | 3.93±0.08 | 37.77±0.26 | 58.70±2.05 | 3.23±0.18 | 1.59±0.04 | 342.00±155.24 | 6.75±0.20 | 28.50±2.75 | 2.70±0.57 | 16.27±0.12 |

WBC: White blood cells. Neu%: Percentage of neutrophils. Lym%: Lymphocyte percentage. Mon%: Monocyte ratio. Bas%: Percentage of basophils. PLT: Platelet. MPV: Mean platelet volume. MFR: Medium fluorescence intensity, reticulocyte percentage. HFR: Percentage of reticulocytes with high fluorescence intensity. RHE: Antigen E factor is present in the RH blood group system in vivo

| Species | Gene | Primer sequence | Tm |
| --- | --- | --- | --- |
| Sheep -L | HBB-F | ATGCTGACTGCTGAGGAGAAGGCTT | 64℃ |
|  | HBB-R | TTAGTGATATCTGTGGGCCAGGGCA |  |
|  | HBA-F | ATGGTGCTGTCTGCCGCCGACAAGG | 64℃ |
|  | HBA-R | TTAACGGTATTTGGAGGTCAGCACG |  |
| Sheep -H | HBB-F | ATGCTGACTGCTGAGGAGAAGGCTT | 64℃ |
|  | HBB-R | TTAGTGATATCTGTGGGCCAGGGCA |  |
|  | HBA-F | ATGGTGCTGTCTGCCGCCGACAAGG | 64℃ |
|  | HBA-R | TTAACGGTATTTGGAGGTCAGCACG |  |
| Pig-L | HBB-F | ATGGTGCATCTGTCTGCTGAGGAGA | 66℃ |
|  | HBB-R | TTAGTGGTACTTGTGGGCCAGGGCA |  |
|  | HBA-F | ATGGTGCTGTCTGCCGCCGACAAGG | 64℃ |
|  | HBA-R | TTAACGGTATTTGGAGGTCAGCACG |  |
| Pig-H | HBB-F | ATGGTGCATCTGTCTGCTGAAGAGA | 64℃ |
|  | HBB-R | TTAGTGGTACTTGTGGGCCAGGGCA |  |
|  | HBA-F | ATGGTGCTGTCTGCCGCCGACAAGG | 64℃ |
|  | HBA-R | TTAACGGTATTTGGAGGTCAGCACG |  |
| Cattle | HBB-F | ATGCTGACTGCTGAGGAGAAGGCTG | 64℃ |
|  | HBB-R | TTAATGATATCTGTGGGCCAGGGCA |  |
|  | HBA-F | ATGGTGCTGTCTGCCGCCGACAAGG | 64℃ |
|  | HBA-R | TTAACGGTATTTGGAGGTCAGCACG |  |
| Yak | HBB-F | ATGCTGACTGCTGAGGAGAAGGCTG | 66℃ |
|  | HBB-R | TTAATGATATCTGTGGGCCAGGGCA |  |
|  | HBA-F | ATGGTGCTGTCTGCCGCCGACAAGG | 64℃ |
|  | HBA-R | TTAACGGTATTTGGAGGTCAGCACG |  |
| Rabbit | HBB-F | TCTCCTCACTGGACAGATGCACCAT | 66℃ |
|  | HBB-R | TCAGTGGTATTTGTGAGCCAGGGCA |  |
|  | HBA-F | ATGGTGCTGTCTCCCGCTGACAAGG | 64℃ |
|  | HBA-R | TTAACGATACTTGGAGGTGAGCACG |  |
| Pika | HBB-F | ATGGTGCACCTTTCCGGTGAGGAGA | 66℃ |
|  | HBB-R | TTAGTGGTACTTGTGAGCCAGGGCA |  |
|  | HBA-F | ATGGTGCTGTCTCCCGCTGACAAGG | 64℃ |
|  | HBA-R | TTAACGATACTTGGAGGTGAGCACG |  |
| Mouse | HBB-F | ATGGTGCACCTGACTGATGCTGAGA | 64℃ |
|  | HBB-R | TTAGTGGTACTTGTGAGCCAGGGCA |  |
|  | HBA-F | ATGGTGCTCTCTGGGGAAGACAAAAG | 66℃ |
|  | HBA-R | TTAACGGTACTTGGAGGTCAGCACGG |  |
| Vole | HBB-F | ATGGTGCACCTGACTGATGCCGAGA | 66℃ |
|  | HBB-R | TTAGTGGTACTTGTGAGCCAGGGCA |  |
|  | HBA-F | ATGGTGCTCTCTGGGGACGACAAGAC | 64℃ |
|  | HBA-R | TTAACGGTACTTGGAGGTCAGCACGG |  |

**Table S3 Primer information for plasmid construction of HBB and HBA from animals in high-altitude Tibetan species and their low-altitude counterparts**

**Table S4 Primer information for qPCR of HBB/HBA from different species and the average number of HBB/HBA plasmids within a single cell**

| Species | Gene | Primer sequence | Tm |
| --- | --- | --- | --- |
| Sheep -L | HBB-F | TCCTCTGCTGATGCTGTTATGAA | 55℃ |
|  | HBB-R | CCAGGAGCCTGAAGTTCTCA |  |
|  | HBA-F | CCGACAAGGGCAATGTCAAG | 55℃ |
|  | HBA-R | TAGGTCTTGGTGGTGGGGAA |  |
| Sheep -H | HBB-F | AACGTGGAAGAAGTCGGTGG | 55℃ |
|  | HBB-R | GGGTTGCCCAAAATGGCATC |  |
|  | HBA-F | CCGACAAGGGCAATGTCAAG | 55℃ |
|  | HBA-R | TAGGTCTTGGTGGTGGGGAA |  |
| Pig-L | HBB-F | CTGGTTGTCTACCCCTGGAC | 55℃ |
|  | HBB-R | GGCCTTCACCTTAGCGTTGT |  |
|  | HBA-F | GCAGAGGCCCTGGAAAGAAT | 55℃ |
|  | HBA-R | GGGGTTGAAATCATCGGGGT |  |
| Pig-H | HBB-F | ATGGTGCATCTGTCTGCTGAA | 55℃ |
|  | HBB-R | TCACCACCAACTTCGTCCAC |  |
|  | HBA-F | GCAGAGGCCCTGGAAAGAAT | 55℃ |
|  | HBA-R | GGGGTTGAAATCATCGGGGT |  |
| Cattle | HBB-F | ACTTGTCCACTGCTGATGCT | 55℃ |
|  | HBB-R | CATCGAGATGCTTCATGCCATT |  |
|  | HBA-F | TTCCCCACCACCAAGACCTA | 55℃ |
|  | HBA-R | GAAATCACTGGGGAGGTGGG |  |
| Yak | HBB-F | TCACCGCCTTTTGGGGCAA | 55℃ |
|  | HBB-R | GCACCGGGGTGAATTCCTTG |  |
|  | HBA-F | CCGACAAGGGCAATGTCAAG | 55℃ |
|  | HBA-R | AGGTCGAAGTGGGGGAAGTA |  |
| Rabbit | HBB-F | TCTTCGAGTCCTTTGGGGAC | 55℃ |
|  | HBB-R | TGTCCAGGTGATTCAGACCC |  |
|  | HBA-F | AAGGCCCACGGCAAGAAG | 55℃ |
|  | HBA-R | CAGTGAGCCAGGAGCTTGAA |  |
| Pika | HBB-F | TTTGATTCCTTTGGTGATCTGAGC | 55℃ |
|  | HBB-R | CTTGAGGCTGTCCAGGTGAT |  |
|  | HBA-F | TGACAAGGCCAACGTCAAGG | 55℃ |
|  | HBA-R | TCGAAGTGGGGGAAGTAGGT |  |
| Mouse | HBB-F | CTTGGACCCAGCGGTACTTT | 55℃ |
|  | HBB-R | ATGCAGCTTGTCACAGTGGA |  |
|  | HBA-F | GCTGAAGCCCTGGAAAGGAT | 55℃ |
|  | HBA-R | GGAGCTTGAAGTTGACGGGA |  |
| Vole | HBB-F | ACCTGACTGATGCCGAGAAG | 55℃ |
|  | HBB-R | GGACAGGTCCCCAAAGTGTT |  |
|  | HBA-F | ATCAAGAATGCCTGGGGCAA | 55℃ |
|  | HBA-R | TGGGGTATGCCACGAACATC |  |
|  | tdTomato-F | ACCACCTGTTCCTGGGGC | 55℃ |
|  | tdTomato-R | GATGACGGCCATGTTGTTGTC |  |
| Human | HBB-F | AAGTCTGCCGTTACTGCCCT | 55℃ |
|  | HBB-R | AGCCTTCACCTTAGGGTTGC |  |
| Mouse  (housekeeping gene) | Rpl19-F | GACGGAAGGGCAGGCATATG | 55℃ |
|  | Rpl19-R | TGTGGATGTGCTCCATGAGG |  |

**Table S5 Number of Lin^-^ cells from donor mice for transplantation models establishment**

| Number of donor mice | Number of Lin^-^cells | Number of recipient mice (death) -0d | Number of recipient mice (death) -20d |
| --- | --- | --- | --- |
| 1 | 1×10^7^ | 20 (1) | 19 (0) |
| 2 | 2×10^7^ | 20 (2) | 18 (0) |
| 3 | 3×10^7^ | 20 (1) | 19 (0) |
| 4 | 4×10^7^ | 20 (4) | 15 (1) |

**Table S6 Primer information of genes for qPCR validation the transcriptome sequencing**

| Gene | Primer sequence | Tm |
| --- | --- | --- |
| HK1 | F:CCGAGCTGAAGGATGACCAA | 55℃ |
|  | R:CGTCATAGTCCCCTCGCTTC |  |
| Ppp1r15a | F:CAAGGTAGAGGCACACAGGG | 55℃ |
|  | R:TCGATCTCGTGCAAACTGCT |  |
| Rragd | F:CATGAGTGCTGGGAACCAGG | 55℃ |
|  | R:CATCCTGGGAGTCGATGACG |  |
| Ets1 | F:GCCCAGCTTCATCACAGAGT | 55℃ |
|  | R: CTTCCACGACTGGCTCTCC |  |
| Hexa | F:GCACATTTGGACCGGTGAAC | 55℃ |
| Pim1 | R:CCCGCCACACCTGTATGATT  F:TAAATGGTGCCTGTCCCTGA  R:TGGATCTCAGAAGCTGCCTG |  |
|  |  | 55℃ |
|  |  |  |
